# The Plant-Transpiration Response to Vapor Pressure Deficit (VPD) in Durum Wheat Is Associated With Differential Yield Performance and Specific Expression of Genes Involved in Primary Metabolism and Water Transport

**DOI:** 10.3389/fpls.2018.01994

**Published:** 2019-01-15

**Authors:** Susan Medina, Rubén Vicente, Maria Teresa Nieto-Taladriz, Nieves Aparicio, Fadia Chairi, Omar Vergara-Diaz, José Luis Araus

**Affiliations:** ^1^Integrative Crop Ecophysiology Group, Plant Physiology Section, Faculty of Biology, University of Barcelona (UB), Barcelona, Spain; ^2^Facultad de Ciencias Ambientales, Universidad Científica del Sur, Lima, Peru; ^3^National Institute for Agricultural and Food Research and Technology (INIA), Madrid, Spain; ^4^Agricultural Technology Institute of Castilla and León (ITACYL), Valladolid, Spain

**Keywords:** durum wheat, gene regulation, drought, transpiration, yield, water transport, vegetation indices, aquaporin

## Abstract

The regulation of plant transpiration was proposed as a key factor affecting transpiration efficiency and agronomical adaptation of wheat to water-limited Mediterranean environments. However, to date no studies have related this trait to crop performance in the field. In this study, the transpiration response to increasing *vapor pressure deficit* (VPD) of modern Spanish semi-dwarf durum wheat lines was evaluated under controlled conditions at vegetative stage, and the agronomical performance of the same set of lines was assessed at grain filling as well as grain yield at maturity, in Mediterranean environments ranging from water stressed to good agronomical conditions. A group of linear-transpiration response (LTR) lines exhibited better performance in grain yield and biomass compared to segmented-transpiration response (STR) lines, particularly in the wetter environments, whereas the reverse occurred only in the most stressed trial. LTR lines generally exhibited better water status (stomatal conductance) and larger green biomass (vegetation indices) during the reproductive stage than STR lines. In both groups, the responses to growing conditions were associated with the expression levels of dehydration-responsive transcription factors (*DREB*) leading to different performances of primary metabolism-related enzymes. Thus, the response of LTR lines under fair to good conditions was associated with higher transcription levels of genes involved in nitrogen (*GS1* and *GOGAT*) and carbon (*RCBL*) metabolism, as well as water transport (*TIP1.1*). In conclusion, modern durum wheat lines differed in their response to water loss, the linear transpiration seemed to favor uptake and transport of water and nutrients, and photosynthetic metabolism led to higher grain yield except for very harsh drought conditions. The transpiration response to VPD may be a trait to further explore when selecting adaptation to specific water conditions.

## Introduction

Agriculture is highly vulnerable to climate change, which is expected to modify crop productivity. A predicted rise in ambient temperatures, together with a decrease in precipitation will likely increase the severity and frequency of drought stresses in the Mediterranean basin, which will negatively affect crop performance (Li et al., 2009; Ceccarelli et al., 2010). Durum wheat is one of the most important crops in Mediterranean countries due to its use as a staple food (IGC, 2017, http://www.igc.int/es/). Additional efforts are required to increase yield gains for the coming decades by selecting traits for higher productivity under high temperatures and water limitation (Robertson et al., 2016). In that sense, a combination of classical and novel breeding approaches, together with the choice of the proper phenotyping traits and a better understanding of the complex metabolic mechanisms operating under abiotic stresses may contribute to that aim (Araus et al., 2008; Tardieu et al., 2011; Mwadzingeni et al., 2016). Most traits of agronomic significance are complex and controlled by multiple genes and environmental signals that determine plant phenotype (Ficklin and Feltus, 2013). Therefore, improvement in yield production under stress (e.g., drought) conditions may benefit from an integrative approach, combining different levels (organ, individual plants, crop) of phenotyping, together with molecular characterization (Liu and Able, 2017).

Mediterranean environments are characterized by water scarcity that usually develops during spring, which in the case of durum wheat (and other small grain cereals) coincides with the grain filling period. Therefore, an increase in grain yield does not necessarily require crops with a higher water use efficiency but instead with a more effective use of water (Blum, 2009). This concept refers not only to the photosynthetic activity of the plant but also to its capacity to manage the amount of water that is available in the soil in order to sustain plant transpiration, particularly under water limited environments (Lopes et al., 2011). The transpiration rate of the plant is driven by changes in vapor pressure deficit (VPD), which is a combined function of air temperature and relative humidity (Kholová et al., 2012; Belko et al., 2013) and is calculated as the saturated vapor pressure minus the actual vapor pressure. Recently, Lobell et al. (2014) have reported for maize that atmospheric VPD, also termed ‘atmospheric drought’, has a much stronger effect on current and future yields than previously thought. Moreover, transpiration responses to increasing VPD have been linked both theoretically and experimentally to yield under terminal water deficit regimes (Vadez et al., 2014). This is relevant for cereals under Mediterranean conditions, which are exposed to terminal (i.e., during grain filling) droughts. Furthermore, the increase in the frequency of heat and drought events in the Mediterranean, driven by climate change, will result in stronger VPD conditions.

In wheat, large genetic variability has been reported in transpiration sensitivities to evaporative demand and leaf areas (Schoppach and Sadok, 2013). Recently, Schoppach et al. (2017) have studied whole plant transpiration in cultivars representing 120 years of breeding for the Mediterranean conditions of southern Australia. Thus, selection over time by breeders for yield increase has unconsciously resulted in genotype selection for the expression of the limited-transpiration trait. These authors conclude that limited (or segmented) whole-plant transpiration under high atmospheric VPD has resulted in advantageous water conservation and crop yield increase under the particular Mediterranean conditions of Australia. Moreover, changes in transpiration rates were independent of plant leaf area and only marginally correlated with phenology (Schoppach et al., 2017). However, other evidence from wheat (Schoppach and Sadok, 2013; Schoppach et al., 2016) and soybean (Devi et al., 2016) suggests that transpiration rates and leaf area responses to VPD are coupled such that increases in transpiration under high VPD are “compensated” by decreases in leaf area. This suggests the existence of a trade-off between both traits that may eventually diminish or even offset the potential usefulness when breeding for transpiration rates. In any case, studies in species other than wheat also indicate that limited transpiration at high VPD in water-limited environments results in yield increases (Gholipoor et al., 2010). However, the environments typical of the Mediterranean climate conditions of Australia are drought prone, with wheat yields frequently below 3 tons per hectare (Robertson et al., 2016).

Besides the need to investigate the potential consequences at the agronomical level of the genotypic differences in transpiration response to VPD, more effort is required to understand the mechanisms underlying the genotypic responses of this trait (Vadez et al., 2014). A recent study in bread wheat using a mapping population composed of 143 DH lines grown in greenhouse conditions identified six QTLs for the transpiration response to VPD, with one major QTL harboring several genes previously reported as being involved in ABA signaling, interactions with *DREB2A* and root hydraulics (Schoppach et al., 2016). Genetic differences in the response of transpiration seem to also have a hydraulic basis, in which aquaporins might play a role (Vadez et al., 2014). Similarly, in pearl millet, limitations in transpiration demand in a high VPD environment were genotype-specific, linked to drought adaptation mechanisms involving abscisic acid and hydraulic signals (Kholová et al., 2010a; Kholová and Vadez, 2013). However, to the best of our knowledge there are no studies in wheat relating the phenotyping characteristics of plant transpiration under increasing VPD to agronomical and physiological performance and gene expression under field conditions in the same lines.

On the other hand, many transcription factors and stress-inducible genes have been identified under drought conditions (Table 1). These include dehydration-responsive element-binding proteins and dehydrins with protective functions in stress (e.g., drought) conditions (Salekdeh et al., 2009; Gahlaut et al., 2016; Yousfi et al., 2016). In addition, to counteract the increased levels of reactive oxygen species under water stress, genes related to protective functions are generally overexpressed. Also relevant under water stress, are the movements of water and other small solutes such as CO_2_, ammonia and urea, which are mediated by water channel proteins known as aquaporins. In addition, water stress affects the main metabolic pathways and regulatory mechanisms. This leads to a downregulation of genes involved in photosynthesis, N uptake and assimilation, amino acid synthesis, and upregulation in energy provision genes, and also those involved in remobilization and protective functions (Habash et al., 2014; Vicente et al., 2015; Cheng et al., 2016; Medina et al., 2016; Yousfi et al., 2016).

**Table 1:** Transcription factors, stress-inducible genes, and main metabolic pathway genes in wheat.

**Factor**	**Significance**	**Reference**
**TRANSCRIPTION FACTORS, STRESS-INDUCIBLE GENES**		
Dehydration-responsive element-binding proteins, *DREB1* and *DREB2*	Regulators of several developmental mechanisms in the response to stress, including drought.	Salekdeh et al., 2009; Gahlaut et al., 2016; Yousfi et al., 2016
Dehydrins *DHN* and *Wcor719*	Related to cold-responsive and protective functions in stress conditions. Up-regulated under water and cold stress.	Danyluk et al., 1996; Tsvetanov et al., 2000; Talamè et al., 2007; Kosová et al., 2014b
Superoxide dismutase enzyme (*SOD*)	Plays a key role in the elimination of superoxide and prevents cell damage. It has increased levels under water stress and is related to protective functions.	Huseynova et al., 2015
Aquaporins *(PIP and TIP)*	Water channel proteins that belong to the major intrinsic protein superfamily. Relevant under water stress in the movement of water and other small solutes such as CO_2_, ammonia, and urea.	Forrest and Bhave, 2007; Hove et al., 2015
**MAIN METABOLIC PATHWAY GENES**		
ATP synthase (*ATPase*)	Major role in the synthesis of ATP and energy provision to the primary and secondary metabolism.	Zhang et al., 2008; Cheng et al., 2016
Ribulose bisphosphate carboxylase oxygenase—Rubisco (*RBCL, RBCS*)	Main enzyme of carbon plant metabolism, catalyses the first step of CO_2_ fixation and photorespiration.	Nagy et al., 2013; Vicente et al., 2015
Phospho*enol*pyruvate carboxylase (*PEPC*)	Participates in carbon mobilization for primary metabolism.	Nagy et al., 2013; Vicente et al., 2015
Pyruvate kinase (*PK*)	Participates in the provision of carbon skeletons for the biosynthesis of organic and amino acids.	Nagy et al., 2013; Vicente et al., 2015
Chloroplastic glutamine synthetase (*GS2*)	Related to N metabolism, it catalyses the conversion of glutamate into glutamine in the chloroplast.	Nagy et al., 2013; Thomsen et al., 2014; Tian et al., 2015
Cytosolic glutamine synthetase (*GS1*)	Related to N metabolism, it catalyses the conversion of glutamate into glutamine in the cytoplasm.	Nagy et al., 2013; Zhang et al., 2017
Glutamate synthase (*GOGAT*)	Related to the synthesis of glutamate, its activity is related to GS1 and GS2.	Vicente et al., 2015

The aim of this study was to assess the differences in the whole-plant transpiration response to VPD in a set of 20 modern (semi dwarf durum) wheat cultivars widely grown in Spain during the past four decades. Several categories of lines that exhibited different transpirative responses were determined. Further, the agronomical responses of these different subsets of lines in terms of grain yield and physiological characteristics were evaluated under a wide range of environmental conditions provided by different locations, years and water regimes (rainfed and support irrigation). Besides grain yield, crop growth was evaluated using vegetation indices acquired at the plot level (Vergara-Díaz et al., 2016) and the water status were assessed through the analysis of the stable carbon isotope composition of mature kernels (Araus et al., 2013). Finally, differences in the pattern of gene expression during grain filling were investigated in the same set of lines. Thus, differences in transcript profiles for a wide range of genes involved in assimilatory metabolism and defense mechanisms, and between groups of linear and segmented transpiration lines, were evaluated under contrasting water regimes (rainfed vs. irrigation). The results obtained may pinpoint future research directions to support breeding programs for selection of lines better adapted to future climate scenarios.

## Materials and Methods

### Experimental Setup

Two different groups of experiments were carried out. The first was under controlled conditions to study differences among lines in transpiration patterns under increasing VPD. The second was under field conditions, during two consecutive crop seasons, to assess genotypic variability in grain yield and physiologically related parameters in a wide range of environmental conditions, including different water regimes (rainfed vs. support irrigation) and locations (with different temperatures and evaporative demand) in Spain. The agronomic, physiological and molecular measurements performed in the two groups of experiments are summarized in Supplementary Table S1.

The experiments were conducted with a collection of 20 commercial semi-dwarf durum wheat [*Triticum turgidum* L. ssp. *durum* (Desf.)] lines released in Spain during the last four decades (i.e., after Post-Green Revolution): Mexa, Vitron, Simeto, Regallo, Gallareta, Bolo, Don Pedro, Sula, Bólido, Iride, Dorondón, Burgos, Claudio, Amilcar, Pelayo, Avispa, Don Sebastián, Don Ricardo, Kiko Nick, and Ramirez. These cultivars represented high-yielding lines at the time they were released and some of them are still cultivated across the Mediterranean basin. They represent a comprehensive set of the durum wheat germplasm cultivated in Spain after the Green Revolution (Chairi et al., 2018); see details of the lines in Supplementary Table S2.

### Transpiration Response to Vapor Pressure Deficit

The experiment was conducted from August to October 2015 at the Experimental Facilities of the Faculty of Biology at the University of Barcelona. For each line, ten plants were grown in a greenhouse; two seeds were sown in 2 L pots (a total of five pots per line; i.e., five biological replicates) containing a mixture (1:1, v/v) of standard substrate and perlite. Photosynthetic photon flux density (PPFD) at midday of a sunny day inside the greenhouse was 800 μmol m^−2^ s^−1^, the average day/night temperature 25/17°C and the relative humidity (RH) 50%. The plants were uniformly irrigated every 2 days with 50% Hoagland's nutrient solution (Hoagland and Arnon, 1950). At 36 days after sowing, corresponding to Zadoks stage 23–25 (Zadoks et al., 1974), plants were fully irrigated to reach 100% pot capacity and drained overnight. During the afternoon of the next day, all pot surfaces were completely covered with a layer of aluminum foil to avoid evaporation, and transferred to controlled environment chambers (Conviron E15; Controlled Environments, Winnipeg, MB, Canada) for acclimatization with a night temperature of 15°C and 70% RH (with a night VPD of 0.51 kPa). The following day the transpiration response to changes in VPD was performed by exposing the plants, organized in a complete randomized design, to controlled increments in VPD from 0.6 to 4.1 kPa, applied by changing both temperature and humidity every hour from 8 a.m. (19°C and 70% RH), after 80 min. of light adaptation, to 5 pm (38°C and 40% RH), and maintained at a constant PPFD of ~400 μmol m^−2^ s^−1^ during the entire experiment, as reported in previous studies (Gholipoor et al., 2013; Vadez et al., 2014; Medina et al., 2017). The RH and temperature were recorded by two external sensors (DO9847, Delta Ohm, Caselle di Selvazzano, Italy) placed inside the chamber. Meanwhile, plant transpiration was recorded by weighing each pot every hour on a 10 Kg electronic bench balance with a resolution of 0.1 g (KB Kern 573, Kern & Sohn GmbH, Balingen, Germany); then one transpiration value was recorded per pot at each VPD point based on the loss of pot mass. Further, the plants were harvested by cutting the stem 1 cm above the soil level, and the leaf area was measured immediately by scanning each leaf (HP Scanjet 200, Hewlett-Packard, California, US) and processing the image with Image J software (https://imagej.nih.gov/ij/). To rule out the effect of plant size variation, for each plant the transpiration was normalized to its corresponding leaf area, all transpiration and biomass measures were recorded in the five biological replicates mentioned above.

### Field Trials

The field experiments were carried out from November to June during two consecutive experimental campaigns, 2013–2014 and 2014–2015, at three experimental field locations located in the northern, central, and southern parts of Spain; for growing season details see Table 2. Two water regimes were imposed in the Aranjuez and Valladolid trials (rainfed and supported irrigation), whereas in Seville the plants were evaluated, during the second crop season alone, under rainfed conditions due to the shallow water table from the proximity of the Guadalquivir River to the trial (~0.5 km). Therefore, nine field trials that considered location, water regime and crop season were conducted with a completely randomized split plot model with three sets of plot replicates per line and trial. Each plot consisted of six rows 7 m long and 0.2 m apart, with a planting density of 250 seeds m^−2^. During both experimental campaigns the fertilization was applied in two steps, a first basal application and then a second top dressing application (Table 2). All trials were controlled for weeds, insect pests, and diseases by recommended chemical doses (Sanchez-Bragado et al., 2014). Plants were harvested mechanically at maturity and grain yield assessed.

**Table 2:** Field experimental trial conditions.

	**Instituto Tecnológico Agrario de Castilla y León (ITACYL)**	**Instituto Nacional de Investigación Agraria y Alimentaria (INIA)**	
Station	**Valladolid**	**Aranjuez**	**Seville**
Location	Zamadueñas	Colmenar de Oreja	Coria del rio
Latitude	41°41'N, 04°42'W	40°04'N, 3°31'W	37°14'N, 06°03'W
Altitude	700 m a.s.l	590 m a.s.l.	5 m a.s.l
Soil (Organic matter)	Loam (0.8%)	Clay-loam (0.5%)	Loam (0.9%)
**1st Crop season**	2013–2014	2013–2014	
Sowing date	November 25th, 2013	November 22nd, 2013	
Harvesting date	July 22th, 2014	July 9th, 2014	
Conditions	−2 to 26°C/34–99 RH%	0 to 25°C/ 31–95% RH	
Rainfall	212 mm	203 mm	
Supplemented irrigation	125 mm	180 mm	
1st Fertilization:	300 kg ha^−1^	400 kg ha^−1^	
Prior sowing	8:15:15 NPK	15:15:15 NPK	
2nd Fertilization:	300 kg ha^−1^	150 kg ha^−1^	
Top dressing	Calcium ammonium nitrate	Diluted urea (46%)	
Sampling date	May 14th	May 12th	
**2nd Crop season**	2014–2015	2014–2015	2014–2015
Sowing date	November 24th, 2014	November 20th, 2014	December 1st, 2015
Harvesting date	July 10th, 2015	July 22nd,2015	July 10th, 2015
Conditions	4 to 17°C/53–100 RH%	5 to 21°C/27–100 RH%	4 to 28°C/34–99 RH%
Rainfall	258 mm	206 mm	162 mm
Supplemented irrigation	125 mm	180 mm	-
1st Fertilization:	300 kg ha^−1^	400 kg ha^−1^	400 kg ha^−1^
Prior sowing	8:15:15 NPK	15:15:15 NPK	15:15:15 NPK
2nd Fertilization:	300 kg ha^−1^	150 kg ha^−1^	150 kg ha^−1^
Top dressing	Calcium ammonium nitrate	Diluted urea (46%)	Diluted urea (46%)
Sampling date	May 15th	May 13th	April 17th

### Field Measurements, Sampling, and Stable Isotope Analysis

Field measurements and sampling of flag leaves were performed for all the trials at post-anthesis (Zadok stage 72–73) on sunny days at midday (10 a.m.−2 p.m.). Pools of five flag leaves per plot were frozen into liquid nitrogen and stored at −80°C for laboratory analysis during the 2013–2014 experiments (sampling dates are described in Table 2). The normalized difference vegetation index (NDVI) was estimated in each plot using a hand-held portable spectroradiometer (GreenSeeker, NTech Industries, Ukiah, CA, USA), scanning with the sensor held perpendicularly to the canopy and 0.5–0.6 m above. The relative chlorophyll content was measured with a Minolta SPAD-502 chlorophyll meter (Spectrum Technologies, Plainfield, IL, USA) on the adaxial surface of the central segment of the flag leaf blades, recording five flag leaves per plot and then averaging the readings. Similarly, stomatal conductance (g_s_) was measured in two flag leaves per plot using a Decagon SC-1 Leaf Porometer (Decagon Device, Inc., Pullman, WA, USA). The canopy temperature of each plot was measured with an infrared thermometer (PhotoTemp™ MX6™TM, Raytek Corporation, Santa Cruz, USA). Ambient temperature was measured simultaneously above each plot using a thermo-hygrometer (Testo 177-H1 Logger, Germany). Canopy temperature depression (CTD) was then calculated as the difference between canopy temperature and air temperature. The plant anthesis time was counted in days since the sowing date. The vegetation indices were estimated using digital RGB (red-green-blue) pictures taken above the plot, holding the camera at 0.8–1.0 m above the plant canopy in the zenithal plane and focusing near the center of each plot. Pictures were taken with Olympus EM-10 and Nikon D90 digital cameras, with a focal length of 18 and 14 mm, and fields of view (FOV) of 66° 43′ and 46° 51′, during the 2013–2014 and 2014–2015 crop seasons, respectively, with a shutter speed of 1/125 for both cameras. No flash was used and the aperture remained in automatic mode. Photographs were saved in JPEG format with a size of 4608 × 3456 pixels and 4288 × 2848 pixels for the Olympus and Nikon cameras, respectively. Subsequently, pictures were analyzed with open source Breedpix 0.2 software (Casadessús et al., 2007) designed for digital photograph processing, which determines the RGB vegetation indices from the different properties of color (Hue, intensity, saturation, lightness, a^*^, b^*^, u^*^, v^*^, and GA as green area), according to the method of Vergara-Díaz et al. (2016).

At the end of the season the grain was harvested, and the plant height and yield were recorded for each plot. A representative part of the grain pool in every plot was dried in an oven for 48 h at 70°C, and finely powdered. Then 1 mg samples of powder were weighed into tin capsules for measurements of the stable C (^13^C/^12^C) ratio, together with the total C and N content. Measurements were carried out in an elemental analyser (Flash 1112 EA; ThermoFinnigan, Bremen, Germany) coupled with an isotope ratio mass spectrometer (Delta C IRMS; ThermoFinnigan), operated in continuous flow mode, at the Scientific Facilities of the University of Barcelona as described elsewhere (Bort et al., 2014). The values of each of the above agronomical and physiological traits for each of the 20 lines at a given trial were the average of three different plots.

### Quantitative Reverse Transcriptase PCR Amplification

The frozen flag leaf samples from five plants per plot were ground with liquid nitrogen and subsequently RNA was isolated from 100 mg of this material using Ribozol RNA Extraction Reagents (Amresco, Solon, OH, USA) as described in Medina et al. (2016). RNA quantity was measured by Qbit fluorometric quantification (Qubit™ 3.0 Fluorometer, Thermo Fisher Scientific, Waltham, MA, USA), while RNA integrity was assessed with an RNA bioanalyser (Agilent 2100 Bioanalyzer, Agilent Technologies, Waldbronn, Germany), obtaining RIN (RNA Integrity Number) scores higher than 6.5 for all samples. Total RNA (1 μg) was treated with PerfeCTa DNase I RNase-free (Quanta Biosciences, Gaithersburg, MD, USA) to eliminate residual genomic DNA, and cDNA was synthesized using a qScript cDNA Synthesis Kit (Quanta Biosciences) following the manufacturer's instructions. The qRT-PCR assays, thermal profile and primer design were performed according to Medina et al. (2016). Three technical replicates were analyzed per biological (i.e., plot) replicate and for each primer pair, while primer efficiency and specificity were checked experimentally. The primers used for gene expression analysis are listed in Supplementary Table S3. These included the housekeeping or control genes encoding ubiquitin and the 18S ribosomal subunit, which were used to normalize qRT-PCR results, and which have been used widely in previous reports (Vicente et al., 2015; Yousfi et al., 2016). The target genes investigated encoded the transcription factors *DREB1* and *DREB2*, the dehydrins *Td16* (*DHN16*) and *WCOR719* (*WCOR*), superoxide dismutase (*SOD*), chloroplastic ATP synthase β-subunit (*ATPase*), cytosolic (*GS1*), and chloroplastic (*GS2*) glutamine synthetases, ferredoxin-dependent glutamate synthase (*GOGAT*), phospho*enol*pyruvate carboxylase (*PEPC*), pyruvate kinase (*PK*), the Rubisco large subunit (*RBCL*), and aquaporin *TIP1.1*. The relative expression was analyzed using the comparative C_t_ method (Schmittgen and Livak, 2008) as the change between the expression of the target and reference genes (ΔC_t_) using the fold expression E^−Δ*Ct*^, where E is the corrected efficiency of each primer. For the comparison within categories of lines or environments, gene expression was described in E^−Δ*ΔCt*^ values. Then the target gene relative expression was calculated for each group of lines in higher yield (HY, above 4,000 kg ha^−1^) and lower yield (LY, below 4,000 kg ha^−1^) scenarios. The expression values for each gene in every one of the 20 lines within atrial were the average of three different biological replicates (i.e., plots).

### Data Analysis

To fit the data collected for transpiration rate (TR) and VPD levels, we applied a segmented linear regression (model Y1 = Slope1 × X + Intercept1 and Y2 = Slope2 × X + Intercept2) or a linear regression (model Y1 = Slope1 × X + Intercept1) with 1,000 interactions; these algorithms fitted the better model depending of the data, accounting for a 95% confidence interval and significance of *p* > 0.05, and the slopes were compared. This analysis was performed with GraphPad Prism software (Graph Pad Software Inc, La Jolla, USA). The slope variation (Δslope) was calculated and used to classify the lines according to its sensitiveness to increasing VPD. In addition, the slope of the linear increase in transpiration as VPD was augmented from around 1 to 4 kPa was also calculated to compare with the range of VPD values usually tested in wheat (Schoppach et al., 2016, 2017). In that case, the starting point corresponded to the second measurement, 140 min after the light period started, when VPD reached a value of 1.07 kPa.

The effects of the transpiration response and growing conditions on agronomic, physiological, and gene expression were evaluated through analysis of variance (ANOVA) and linear model comparisons (*p* < 0.001). For gene expression data in particular, a log_2_ transformation was needed. When the differences between treatments were significant (*p* < 0.05), the mean comparison was assessed by LSD (least significant differences). The correlation analysis was performed with the Pearson method (*p* < 0.001). All tests were performed with the R package for statistical computing (R Foundation for Statistical Computing: Vienna, Austria). Heat maps of relative gene expression were generated using a log transformation of the real-time PCR data presented as ΔC_T_ (C_T__mRNA_-C_T__18SrRNA, UBImRNA_) with GraphPad Prism version 7.00 (GraphPad Software, La Jolla California USA). The network analyses for all traits were carried out using significant correlations (*p* < 0.001) with higher Pearson's coefficients (*r* > 0.8 and *r* < −0.8), then the representation was performed by Cytoscape v3.4.0 (Shannon et al., 2003). The Principal Component Analysis (PCA) of the physiological traits and gene expression was performed in R for the LTR and STR+ group of lines.

## Results

### Transpiration Response of Wheat Lines to Changes in Vapor Pressure Deficit

**Figure 1:**
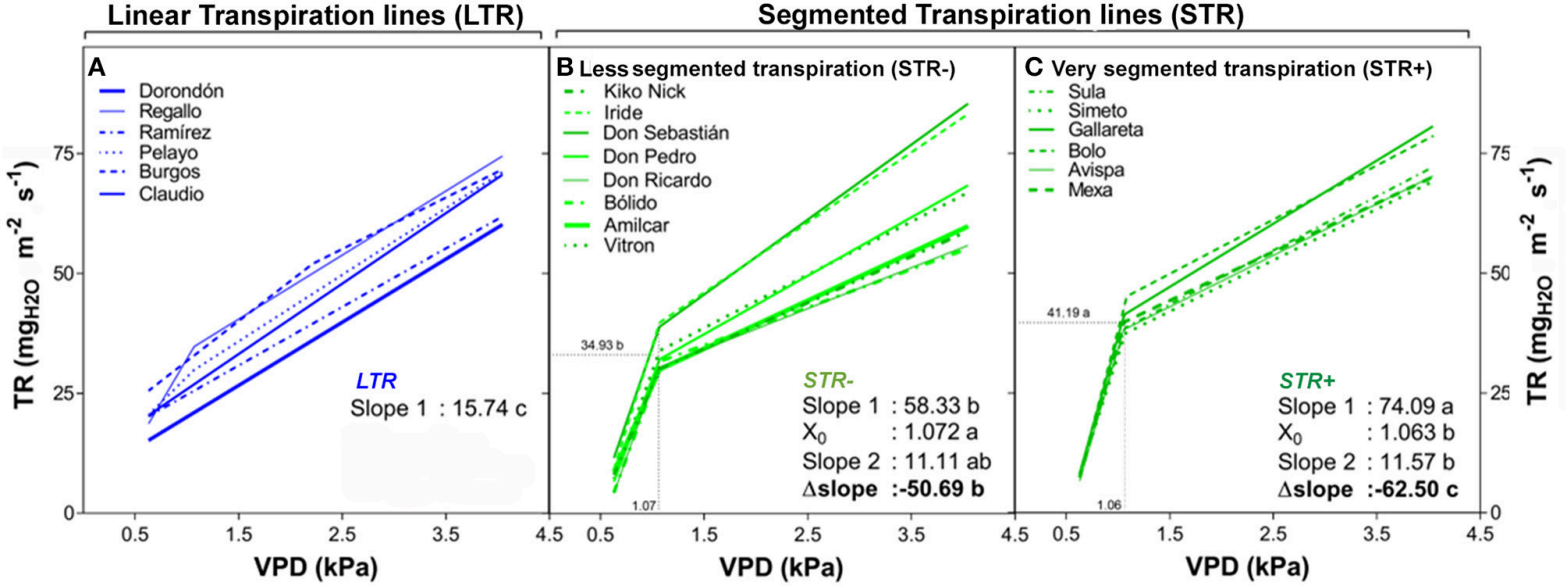
Transpiration rate (TR) of 20 durum wheat lines exposed to increasing VPD regimes as described in Table 3. Plantlets were growth in pots under well-watered conditions in a greenhouse and the transpiration response was tested in a growth chamber as indicated in the Materials and Methods. Each curve expresses the mean TR values across low to high VPD of a particular line. Plants were tested at the vegetative stage and values represent the mean of five plants (i.e., five biological replicates) per line. **(A)** The linear regressions of the linear transpiration lines (LTR), and **(B,C)** the segmented regressions of the segmented transpiration lines [R: less segmented transpiration (STR−) and very segmented transpiration (STR+)] are shown. All panels show the comparison according to the LSD test (*p* < 0.05) indicating the mean slope of the TR response before and after (Slope 1 and 2) putative VPD breakpoints (X_0_) and their slope variation (Δslope).

The transpiration response to increasing VPD under controlled conditions showed significant differences in the slopes of the 20 durum wheat lines in this experiment (Table 3 and Figure 1). The significant variation in the slopes classified the 20 lines into two main groups: those that maintain a linear transpiration response to water loss (LTR), which were not sensitive to VPD changes, and those that showed a segmented transpiration response to water loss (STR), which included lines sensitive to increments in VPD that fell into two different subgroups; less segmented transpiration responses (STR−) and very segmented transpiration responses (STR+). The LTR group included six lines (Burgos, Claudio, Dorondón, Pelayo, Ramirez, and Regallo) that fitted better in a linear regression and did not show a consistent VPD threshold (X_0_) or a higher significant slope variation. The STR- group included the lines Amilcar, Bólido, Don Ricardo, Don Pedro, Don Sebastián, Iride, Kiko Nick, and Vitron and the STR+ group the lines Avispa, Bolo, Gallareta, Mexa, Simeto, and Sula. The slope variation (Δslope) within these three subgroups was significantly different, with values close to zero in LTR lines, and decreasing progressively for STR− and STR+ lines (Table 3). Furthermore, the transpiration started to decrease in both segmented transpiration subgroups at similar VPD break points (STR+: 1.063 and STR−: 1.072 kPa), but the decreases differed significantly in their Δslopes (STR− = −47.2 and STR+ = −62.5 mg_H2O_ m^−2^ s^−1^) (Table 3). The LTR lines showed significant lower mean slopes under low VPD (Slope 1) but higher mean slopes under high VPD (Slope 2) compared to the STR group, whereas STR+ lines showed the highest mean slope value under low VPD and the lowest value under high VPD. The STR- lines showed values between the LTR and STR+ ones. Moreover, the LTR and STR+ groups of lines evidenced clearly significant differences in their transpiration rates in the range of VPD levels from 1 kPa to 3.3 kPa, as shown in Supplementary Figure S1.

**Table 3:** Transpiration response to variations in vapor pressure deficit (VPD) of 20 durum wheat lines.

	**Class**	**Line**	**Intercept**	**Slope 1**	**X_**0**_**	**Slope 2**	**Δslope**	***R^**2**^***
Linear transpiration lines (LTR)	LTR	Burgos	13.89	13.90	-	13.89	0	0.604
	LTR	Claudio	11.11	13.89	-	13.89	0	0.565
	LTR	Dorondón	5.55	11.11	-	11.11	0	0.576
	LTR	Pelayo	8.33	13.89	-	13.89	0	0.718
	LTR	Ramírez	11.11	11.11	-	11.11	0	0.575
	LTR	Regallo	5.55	30.56	1.071	13.89	−16.67	0.403
Segmented transpiration lines (STR)	STR−	Amilcar	−22.22	47.22	1.070	11.11	−36.11	0.814
	STR−	Bólido	−33.33	58.33	1.095	8.33	−50.00	0.881
	STR−	Don Ricardo	−33.33	61.12	1.058	8.33	−52.78	0.811
	STR−	Don Pedro	−30.55	58.33	1.070	11.11	−47.22	0.855
	STR−	Don Sebastián	−27.78	61.11	1.070	16.67	−44.44	0.718
	STR−	Iride	−33.33	69.44	1.070	13.89	−55.56	0.872
	STR−	Kiko Nick	−22.22	50.00	1.070	8.33	−41.67	0.814
	STR−	Vitron	−30.55	61.11	1.070	11.11	−50.00	0.669
		STR−_average_		58.33b	1.072	11.11ab	−50.69a	
	STR+	Avispa	−41.67	75.00	1.054	11.11	−63.89	0.771
	STR+	Bolo	−44.44	80.56	1.086	11.11	−69.44	0.685
	STR+	Gallareta	−38.89	77.78	1.060	13.89	−63.89	0.614
	STR+	Mexa	−41.67	75.00	1.058	11.11	−63.89	0.620
	STR+	Simeto	−33.33	66.67	1.070	11.11	−55.56	0.773
	STR+	Sula	−36.11	69.44	1.070	11.11	−58.33	0.676
		STR+ _average_		74.09a	1.063	11.57b	−62.50	
Linear transpiration lines _average_			9.26a	15.74c	-	12.96a	-	
Segmented transpiration lines _average_			−33.53b	65.08ab	1.069	11.31b	−53.77	

*Plantlets were grown in pots under well-watered conditions in a greenhouse and further the transpiration response was tested in a growth chamber as indicated in the section Materials and Methods. The lines were grouped as linear transpiration lines (LTR) and segmented transpiration lines (STR). The latter was subdivided into lines with less segmented (STR−) or very segmented (STR+) transpiration to water loss, based on the fitted parameters of segmented and linear regressions (p < 0.001) for the transpiration response to changes in VPD between 0.5 and 4.5 kPa. The values represent the mean of five biological replicates. Values with the same letter are not significantly different. The parameters evaluated are the Intercept, the transpiration response at low and high VPD (Slope 1 and 2 respectively), the VPD breakpoint (X_0_), the slope variation between high and low VPD (Δslope), and the R^2^ of the fitting curve. At the bottom is shown the average comparison between LTR and STR lines according the LSD test (p < 0.05), as well as the average comparison between STR− and STR+ inside the STR section. The intercept and Δslope are expressed in mg_H2O_ m^−2^ s^−1^, the X_0_ in kPa and the slopes 1 and 2 in mg_H2O_ m^−2^ s^−1^ kPa^−1^*.

In addition, the transpiration response from 1.07 to 4.10 kPa was also calculated (Supplementary Figure S2 and Supplementary Table S4). For all 20 lines, the relationships between transpiration and VPD were clearly linear and no breakpoint pattern in transpiration was identified as VPD increased. Apart from Burgos and Regallo, all the lines classed as LTR were also among those with the lowest transpiration rates at 1.07 kPa, while their slopes of increase in transpiration in response to increasing VPD were among the greatest. However, Don Sebastián, Iride (classified above as STR- lines), and Gallareta (an STR+ line) were also among the lines exhibiting the greatest slopes, whereas Amilcar, Bólido, Don Ricardo, and Kiko Nick (STR− lines) exhibited relatively low transpiration values at 1.07 kPa.

### Effect of Water Regime and Genotypic Variability on Plant Growth and Yield Associated With Plant Water Lose Management

As expected, lower grain yields for the 20 wheat lines were observed in the rainfed relative to supported irrigation trials (Table 4 Low Yields and High Yields sections). In general, vegetation indices (which are indicators of canopy photosynthetic biomass and leaf greenness) including the spectroradiometrical index Normalized Difference Vegetation Index (NDVI), and the digital photography-derived indices of Hue, lightness, v^*^, and green area (GA) and chlorophyll content (measured at post-anthesis) increased as agronomical conditions improved, while saturation, a^*^ and u^*^ decreased and intensity and b^*^ (which are biomass indicators as well as NDVI) were not affected by growing conditions (Table 4 Low-Medium Yields and High Yields sections). The indicators of water status such as carbon isotope composition (δ^13^C), canopy temperature depression (CTD) and leaf stomatal conductance (g_s_) were also evaluated. Thus, grain δ^13^C decreased and CTD and flag leaf g_s_ increased as growing conditions improved. Finally the N content in grains, which is an indicator of the capacity of the grains to accumulate nitrogen, decreased as growing conditions improved.

**Table 4:** Differences in grain yield, vegetation indices and water regime parameters between the linear transpiration (LTR) and segmented transpiration (STR) groups within each of the nine field growth conditions assayed.

**Low-medium yields**	**Valladolid 2014 rainfed**		**Valladolid 2015 rainfed**		**Aranjuez 2015 rainfed**		**Aranjuez 2015 irrigated**		**Aranjuez 2014 rainfed**		
**Class**	**LTR**	**STR**	**LTR**	**STR**	**LTR**	**STR**	**LTR**	**STR**	**LTR**	**STR**	
Yield	**2668b**	**2851a**	3816	3788	**4759a**	**4621b**	**5127a**	**5019b**	**5893a**	**5378b**	
Int.	0.30	0.31	0.30	0.30	0.32	0.32	0.33	0.33	0.28	0.28	
Hue	70.9	72.7	77.8	77.6	85.1	84.9	86.9	86.2	109.2	110.3	
Sat.	0.32	0.31	0.30	0.29	0.27	0.26	0.28	0.28	0.12	0.11	
Light.	39.5	39.5	39.8	39.8	42.6	42.6	43.3	43.3	34.8	34.3	
a^*^	−10.4	−10.9	−15.5	−15.0	−17.9	−17.7	−19.5	−19.1	−12.2	−11.7	
b^*^	27.1	26.9	26.9	26.3	26.9	26.7	28.3	28.0	14.1	13.4	
u^*^	−2.1	−2.8	−8.9	−8.5	−12.1	−11.9	−13.7	−13.2	−8.7	−8.3	
v^*^	28.5	28.5	29.8	29.2	31.1	30.9	32.7	32.4	16.8	15.9	
GA	0.62	0.64	0.8	0.78	0.98	0.98	0.99	0.99	0.86	0.83	
SPAD	52.3	55.3	51.9	52.4	58.1	58.2	**57.6a**	**59.6b**	**57.1b**	**58.5a**	
NDVI	0.53	0.54	0.66	0.65	0.76	0.76	0.78	0.78	**0.64a**	**0.62b**	
C%	**36.5b**	**43.1a**	43.1	42.9	42.6	42.7	41.3	41.3	41.3	41.7	
δ^13^C	**−23.9b**	**−23.6a**	−24.1	−24.3	**−26.3a**	**−26.5b**	−26.4	−26.5	−25.9	-25.8	
CTD	0.94	1.13	1.52	1.33	7.44	7.54	7.12	6.83	4.74	4.81	
g_s_	190	153	–	–	–	–	–	–	**402a**	**382b**	
N%	**2.2b**	**2.4a**	2.9	2.8	2.4	2.3	2.5	2.5	2.3	2.3	
Flowr.	91.75	92.38	80.82	81.92	92.14	92.05	102.5	102.4	82.26	82.72	
Height	146.7	147.2	146.6	146.7	150.1	149.8	146.8	147.1	151.8	150.9	
**High yields**	**Seville 2015**		**Valladolid 2014 irrigated**		**Aranjuez 2014 irrigated**		**Valladolid 2015 irrigated**		**LTR**	**STR**	
**Class**	**LTR**	**STR**	**LTR**	**STR**	**LTR**	**STR**	**LTR**	**STR**		**STR+**	**STR−**
Yield	**6470a**	**6436b**	**6656a**	**6432b**	**7215a**	**6704b**	**7417a**	**7124b**	**5540a**	**5346b**	**5430b**
Int.	0.31	0.31	0.29	0.29	0.27	0.26	**0.33b**	**0.34a**	0.30	0.30	0.30
Hue	89.7	89.9	98.2	94.7	109.1	104.4	86.3	86.4	**89.7b**	**91.5a**	**88.6b**
Sat.	0.3	0.29	0.19	0.19	0.14	0.16	0.28	0.26	**0.25a**	**0.23b**	**0.24ab**
Light.	42.3	42.2	38.1	37.9	**34.2a**	**32.9b**	**43.5b**	**44.1a**	39.8	39.5	39.5
a^*^	−21.4	−21.1	−16.5	−16.2	**−15.5b**	**−14.4a**	−19.3	−18.6	**−16.5b**	**−15.9a**	**−16.1a**
b^*^	29.6	29.1	20.4	20.9	16.1	16.9	28.3	27.2	**24.4a**	**23.4b**	**24.3ab**
u^*^	−15.7	−15.4	−12.0	−11.5	**−11.5b**	**−10.4a**	−13.4	−12.8	−10.9	−10.6	−10.5
v^*^	33.7	33.3	23.9	24.4	18.8	19.5	32.8	31.9	**27.8a**	**26.7b**	**27.6a**
GA	0.99	0.99	0.91	0.9	0.92	0.94	0.98	0.97	0.90	0.88	0.89
SPAD	53.8	54.1	57.1	55.8	58.6	57.9	51.1	52.5	**55.1b**	**57.0a**	**55.3ab**
NDVI	0.77	0.76	0.68	0.69	**0.74a**	**0.72b**	0.74	0.73	**0.70a**	**0.69b**	**0.69b**
C%	40.7	40.9	42.1	42.9	41.8	41.3	42.7	41.9	**41.4b**	**41.7a**	**42.5a**
δ^13^C	−27.6	−27.6	−24.8	−24.7	−26.4	−26.3	**−26.0b**	**-25.8a**	−25.7	−25.7	−25.6
CTD	5.37	5.44	2.47	2.55	**4.83a**	**4.44b**	4.92	4.96	5.80	6.05	5.60
g_s_	–	–	242	258	455	424	–	–	**322a**	**306b**	**311b**
N%	2.5	2.4	2.6	2.7	2.2	2.2	2.3	2.3	2.4	2.4	2.5
Flowr.	109.5	106.7	71.06	72.3	84.03	83.64	97.43	95.66	89.76	89.76	90.45
Height	110.2	110.7	157	155.7	133.6	133.3	125.8	125.8	141.48	141.481	140.5

Results are distributed as low to medium yield (upper subtable) and high yield (lower subtable). The LTR, STR+ and STR− columns (right side of the lower subtable) show the mean average across all field trials plots for the STR (including less segmented, STR−, and very segmented, STR+, lines) and LTR lines. For each line and field growing condition (i.e., trial) assayed three biological (i.e., plot) replications were considered. Means exhibiting different letters (and highlighted in bold) are significantly different (p < 0.05) by ANOVA and the LSD test. Grain yield (Yield) in kg. ha^−1^; Vegetation indices: intensity (Int.), Hue, saturation (Sat.), lightness (Light.), a

* , b^*^, u^*^, v^*^, GA, SPAD and NDVI; water status/photosynthetic traits: carbon content (C%) in % of dry mass, carbon isotope composition (δ^13^C) in %0, canopy temperature depression (CTD) in °C, stomatal conductance (g_s_) in μmol m^−2^ s^−1^ and nitrogen content (N%) in % of dry mass; anthesis time (Flowr) in days after January 1st; plant height (Height) in cm.

**Figure 2:**
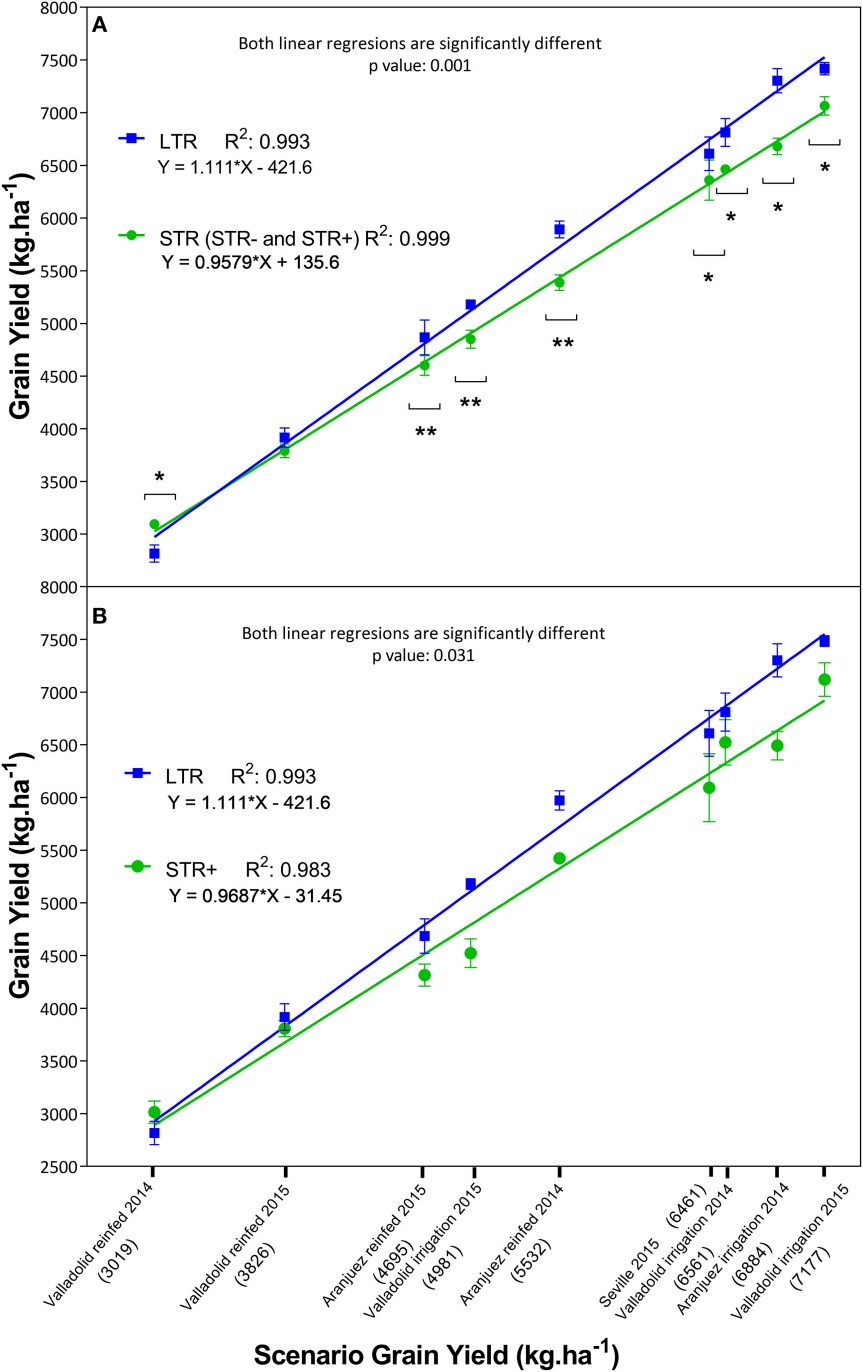
Grain yield differences between the segmented transpiration (STR: including less segmented transpiration, STR−and very segmented transpiration, STR+) and linear transpiration (LTR) lines across nine different growing conditions in the field. The X-axis presents the mean yield values of the complete set of 20 lines within each of the 9 trials where the mean yield across lines is expressed within parentheses below the name of the trial, while the Y-axis exhibits the mean yield of each subset of lines. In **(A)** the linear regressions represent the grain yield of the LTR (blue squares) and STR (green circles) groups within each growing scenario. Asterisks indicate significant differences between the LTR and STR groups performed by ANOVA (^*^p < 0.05, ^**^p < 0.01). In **(B)** the linear regressions represent the average grain yield of the complete set of STR+ and LTR lines. Each value represents the average of any of the two groups of lines, each line being the mean value of three biological replicates (plots) per growing condition (i.e., trial). The level of significance (*p*) between fitting lines as well as the determination coefficient (R^2^) and the equation of each line are also indicated next to each legend.

We observed differences in grain yield and physiological traits between groups, according to their capacity to manage water loss (Table 4 Low-Medium Yields and High Yields sections), and this was evaluated in the same way as the whole-plant transpiration response to increasing VPD under controlled conditions (Table 3 and Figure 1). LTR lines showed different yields compared to STR lines (Figure 2A), except for the trial with the lowest average grain yield (Valladolid rainfed 2014) in which the STR lines exhibited higher yields than the LTR lines, and the second trial with the lowest yield (Valladolid rainfed 2015) in which no differences between STR and LTR lines were recorded. In the other seven trials the LTR lines showed higher yields than STR lines. Good linear fits for grain yield of each group against average grain yield in every growing condition were achieved for each group (${\text{R}}_{\text{LTR}}^{2}$: 0.993 and ${\text{R}}_{\text{STR}}^{2}$: 0.999; Figure 2A), and highly significant differences (*p* < 0.001) between the LTR and STR fitting lines were observed, especially under high-yielding conditions. In addition, the comparison between the linear fit of yield for the most contrasting groups (LTR and STR+) was also significantly different (*p* < 0.031; Figure 2B) as were the transpiration profiles of these groups of lines. Moreover, when the whole set of trials were considered, LTR lines exhibited higher NDVI values and higher RGB indices in some cases (saturation, b^*^, v^*^) and lower values of other indices (Hue, a^*^) plus lower leaf chlorophyll content and carbon content than STR lines (Table 4 High Yields section). Considering the two sub-groups in the STR lines (STR- and STR+) relative to LTR lines, the STR+ lines showed lower values for saturation, b^*^, and v^*^ and higher values for Hue than the STR− and LTR lines. The LTR lines also exhibited slightly higher g_s_ than the STR lines, whereas no clear differences in CTD, δ^13^C, anthesis time, and plant height emerged for the whole set of trials. However, significant differences were observed in grain δ^13^C between the LTR and STR groups within some of the trials. LTR lines exhibited more negative δ^13^C compared to STR lines in the two extreme trials in terms of grain yield (Table 4 High Yields section). Further, the water status, which was represented as the mean grain δ^13^C of the most contrasting groups of lines (STR+ and LTR) against the average δ^13^C value for the whole set of 20 lines in each field trial, was compared (Supplementary Figure S4). Both of the regression curves for LTR and STR+ were significantly different (*p*<0.036), highlighting that higher (less negative) δ^13^C values were achieved for the STR+ than the LTR groups of lines in the environment (trial) with the highest average δ^13^C values (around −24 %0), whereas in the trial with the lowest (i.e., more negative) average δ^13^C (near −28 %0), differences were absent between the two categories of lines.

### Changes in Gene Expression Between Wheat Lines With Different Transpiration Response Patterns With Respect to Grain Yield Productivity

**Figure 3:**
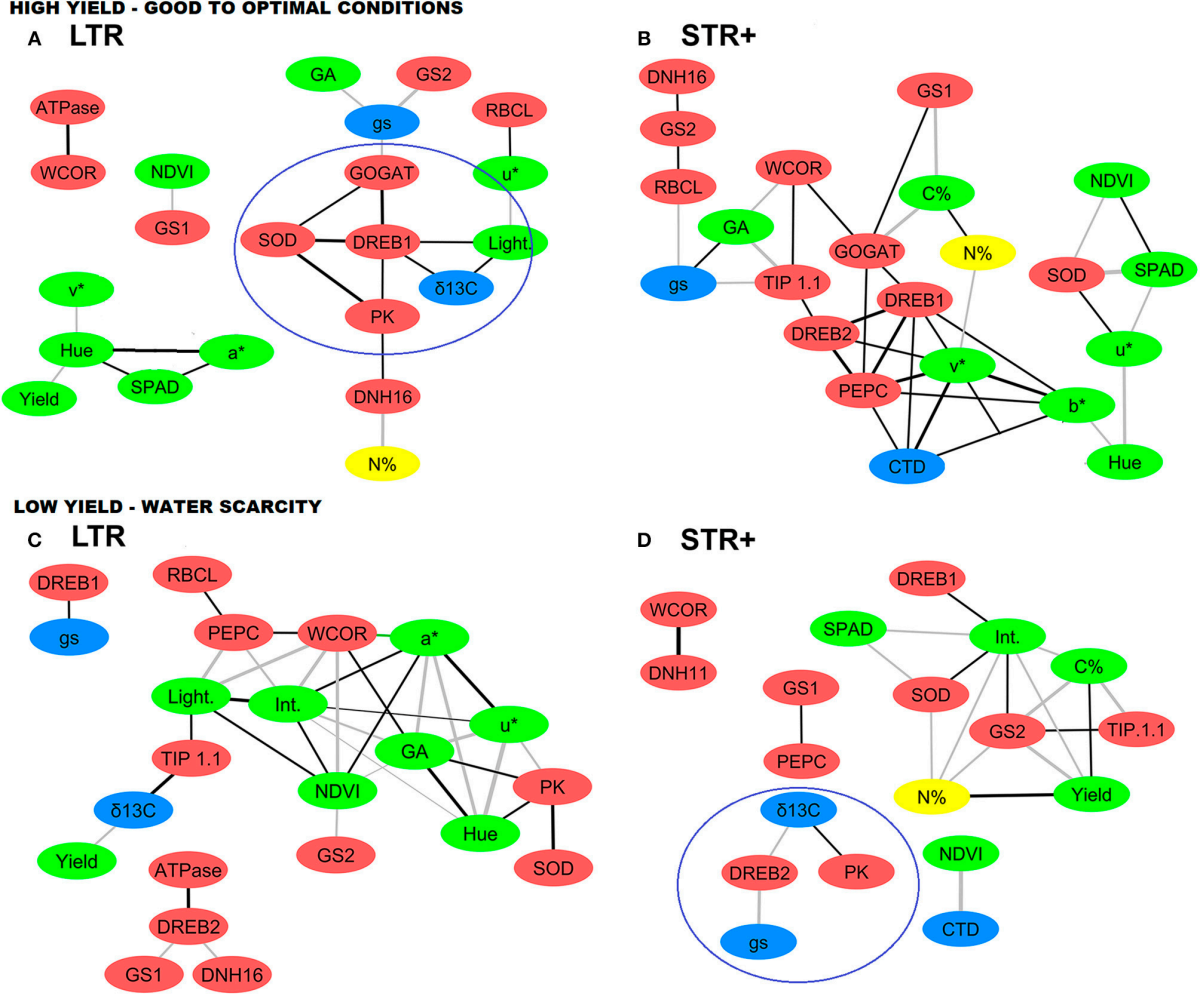
Network analysis of agronomic and physiological traits and transcript levels of very segmented transpiration (STR+) and linear transpiration (LTR) durum wheat lines under **(A,B)** high (HY) and **(C,D)** low (LY) yield environments. Each yield environment includes data of two trials (i.e., growing conditions) at different locations. Red nodes represent transcript levels, green nodes vegetation indices and carbon content, blue nodes water status traits and yellow nodes N content. The black and gray connecting lines (edges) represent significant positive and negative correlations (*p* < 0.05), respectively, based on Pearson's correlation coefficients in which thickness corresponds to the width of the edges (0.7, thinner to 1, thicker). For each of the two groups of lines (STR+ and LTR) with different transpiration patterns, and within a given yield environment (HY and LY), the values used in the network analysis for the different agronomical, physiological and transcription traits were the result of combining all the lines within a transpiration group, with each individual line having three biological (plot) replications per trial. For trait and transcript abbreviations see Tables 4, 5.

The transcript profiles of 13 genes involved in C and N metabolism and the stress response were studied in the 20 wheat lines collected from trials exhibiting strong differences in grain yield associated with the water regime: LY (Valladolid 2014 under rainfed conditions with yield below 4,000 kg ha^−1^) and HY (Valladolid and Aranjuez 2014 under irrigated conditions with yield above 6,000 kg ha^−1^; Figure 3, Table 5). Gene expression analysis indicated significant changes in transcript levels between low and high-yielding scenarios, LTR and STR groups and their interaction. In general terms and compared to the transcript abundance of housekeeping genes (encoding ubiquitin and the 18S ribosomal subunit), the transcript abundances of the *RBCL* and *ATPase* genes were higher, while for the rest of the genes, particularly *DREB1, DREB2, DNH16, WCOR, SOD, GOGAT, GS1, GS2, PEPC, PK*, and *TIP1.1*, they were lower (Table 5). Furthermore, the LTR and STR+ lines showed different profiles within HY and LY while the STR- group showed a gene expression pattern that was intermediate between the other two groups.

**Table 5:** Comparative gene expression of the very segmented transpiration (STR+) and linear transpiration (LTR) lines under low yield (LY, < 3000 kg ha^−1^) and high yield (HY, > 5000 kg ha^−1^) trials assayed in the 2013–2014 experiments.

**Fold change**	**Target gene**	**Line**	**Environment**	**Interaction**	**Low yield (LY)**			**High yield (HY)**		
		**STR+:LTR**	**LY:HY**	**L x E**	**LTR**	**STR+**	**STR+:LTR**	**LTR**	**STR+**	**STR+:LTR**
Stress response	*DREB1*	−3.9	−1.9	^*^	−5.42a	−7.81ab	−5.2	−5.81a	−7.39b	−3.0^**^
	*DREB2*	4.8^*^	−2.1	ns	−8.57b	−8.05a	1.4^*^	−8.24	−5.33	7.5
	*DNH16*	−1.3	−1.9^***^	ns	−4.01	−3.67	1.3	−2.97	−3.90	−2.0
	*WCOR*	2.3	−1.8^*^	ns	−8.01	−7.61	1.3	−7.86	−6.16	3.2
	*SOD*	1.6	−1.8	ns	−6.03	−5.79	1.2	−6.07	−5.10	2.0
N metabolism	*GOGAT*	−1.1^**^	1.2	^*^	−2.87a	−2.91b	−1.0^**^	−3.36c	−3.60c	−1.2
	*GS1*	−13.9^*^	−2.8	ns	−0.66a	−5.40b	−26.8^*^	0.47a	−3.04b	−11.4^*^
	*GS2*	1.3^*^	2.3^**^	ns	−2.65	−2.16	1.4	−3.56b	−3.51a	1.0^*^
C metabolism	*ATPase*	1.0	1.5	ns	0.60	0.69	1.1	−0.18	−0.13	1.0
	*PK*	1.4	−1.9	ns	−5.77	−5.66	1.1	−5.56	−4.77	1.7
	*PEPC*	1.1	−1.6	ns	−6.88	−7.14	0.8	−6.43	−6.13	1.2
	*RBCL*	1.1^*^	1.1	ns	3.63a	2.95b	−1.6^***^	3.11b	3.91a	1.7^*^
Aquaporin	*TIP1.1*	−1.5^**^	3.2^**^	^*^	−2.94a	−3.29b	−1.3^*^	−4.89c	−7.66d	−6.8^***^

The left columns show the fold change in expression of the STR+ lines relative to the LTR lines (Lines column), of LY with respect to HY (Environment column) and their interaction (L x E). Each yield environment (HY and LY) includes data of two trials (i.e., growing conditions) at different locations. For each line and trial assayed three biological (i.e., plot) replications were considered. The Low Yield (LY) and High Yield (HY) columns show the fold change relative to reference genes of the LTR and STR+ groups under HY and LY conditions, as well as the fold change of the STR+ relative to the LTR lines in each environment. The comparisons were assessed by ANOVA and the LSD test using a log_2_ transformation of the fold change values. Different letters indicate significant differences (p < 0.05), while asterisks indicate levels of significance (ns, non-significant;

* p < 0.05;

** p < 0.01;

*** *p < 0.001)*.

*Positive values indicate up-regulation and negative values indicate down-regulation of target genes. For details see section Materials and Methods*.

*DREB1 and DREB2, transcription factors; DNH16, dehydrin; WCOR, actin-binding protein WCOR719; SOD, superoxide dismutase; GOGAT, glutamate synthase; GS1, cytoplasmic glutamine synthetase; GS2, chloroplastic glutamine synthetase; ATPase, ATP synthase; PK, pyruvate kinase; PEPC, phosphoenolpyruvate carboxylase; RBCL, ribulose-1,5-bisphosphate carboxylase oxygenase, large subunit; TIP1.1, aquaporin; N, nitrogen and C, carbon*.

Comparing low yield (LY) relative to high yield (HY) conditions, dehydrin genes (*DNH16* and *WCOR*) were downregulated, whereas the *GS2* and *TIP1.1* genes were overexpressed (Table 5). Considering all growing conditions, STR+ lines overexpressed the *DREB2, GS2*, and *RCBL* genes and underexpressed *GS1, GOGAT*, and *TIP1.1* compared to the LTR group. The expression for the rest of the genes did not reach statistically significant differences between either the subgroups of lines or yielding scenarios. Furthermore, the interaction was significant for *DREB1, GOGAT*, and *TIP1.1*. Comparing STR+ relative to LTR under LY conditions, *DREB2* was significantly overexpressed, whereas *GOGAT, GS1, RBCL*, and *TIP1.1* were downregulated.

### Interaction Network of Physiological Traits and Gene Expression

Four correlation matrices, with a total of 30 variables each, were generated for each combination between contrasting groups of lines (LTR and STR+) and yielding scenarios (HY, LY). Network analysis was performed using significant correlations between parameters based on Pearson correlation coefficients (−0.75 > *r* > 0.75) and *p* values (*p* < 0.05) (Figure 3). The objective was to investigate whether there were changes in gene expression between subsets of lines in association with different agronomical and physiological responses under contrasting yield scenarios.

In the LTR lines under high-yielding conditions (Figure 3A), there were positive relationships (black edges) between the expression of stress responsive genes and other traits (nodes). The target genes comprised: (i) the expression of the transcription factor *DREB1* with the vegetation index (lightness), grain δ^13^C, and the expression of the *GOGAT, PK*, and *SOD* genes; (ii) the expression of dehydrin *DNH16* with N content of grains and the expression of *PK* and (iii) the expression of *WCOR* with *ATPase* expression. With regard to N metabolism, it evidenced negative relationships (gray edges): (i) the expression of *GS1* with biomass (NDVI) and (ii) the expression of both the *GS2* and *GOGAT* genes with g_s_. Concerning C metabolism, the expression of *RBCL* correlated positively with the vegetation index u^*^, as well as the expression of *PEPC* with C content. Vegetation indices also correlated against yield and physiological traits; for example g_s_ correlated negatively with biomass greenness (GA), while Hue correlated positively against grain yield and negatively with chlorophyll content.

For the STR+ lines under high-yielding scenarios (Figure 3B), *DREB1* expression was positively correlated with some vegetation indices (v^*^ and b^*^), as well as with the expression of the *DREB2, GOGAT*, and *PEPC* genes. Both *GS1* and *GOGAT* expression were negatively associated with grain C content, while the expression of *GS2* was positively correlated with *RBCL* and *DHN16* gene expression. *PEPC* expression was positively associated with *DREB2* and *GOGAT* expression and also with some vegetation indices (v^*^ and b^*^). *PK* and *PEPC* expression were positively correlated with δ^13^C and CTD respectively, while the expression of *RBCL* was negatively correlated with g_s_. *TIP1.1* expression was negatively correlated with GA and positively correlated with *DREB2* and *WCOR* expression. Vegetation indices such as NDVI and chlorophyll content exhibited a negative correlation with SOD gene expression.

In LTR lines under low-yielding conditions (Figure 3C), the expression of *DREB2* was positively correlated with *ATPase* gene expression, and negatively correlated with *GS1* and *DNH16* expression. The amount of *WCOR* transcripts was positively correlated with the expression of *PEPC, NDVI* and GA, while the vegetation indices intensity and a^*^ were negatively correlated. *GS2* expression was negatively correlated with biomass (NDVI). *PEPC* expression correlated positively with *RBCL* expression and also with the vegetation index lightness, while the expression of *PK* and *SOD* were positively associated with each other. Grain δ^13^C was negatively associated with yield and positively associated with TIP1.1 expression, while g_s_ was positively correlated to *DREB1* expression. Furthermore, chlorophyll content and grain C content showed a positive correlation between them.

Lastly, for the STR+ lines under low-yielding conditions (Figure 3D) grain yield was positively correlated with grain C and N content, and negatively correlated with the vegetation index intensity and expression of the *GS2* gene. *DREB2* expression was positively correlated with g_s_ and negatively correlated with grain δ^13^C. *GS2* expression was positively associated with *TIP1.1* expression and negatively correlated with grain N and C content, whereas *GS1* expression was positively correlated with *PEPC* expression. Moreover, PK expression and grain δ^13^C were positively correlated.

### Comparative Trait Analysis Between LTR and STR+ Lines

A comparative scheme of gene expression in the primary metabolism pathways and a multivariate PCA analysis performed with field data (Supplementary Figure S4) showed the variation of the physiological and gene expression parameters and their contribution in the groups of lines (LTR and STR+). All biomass (Yield, NDVI, GA and Hue), g_s_, SPAD, and most of gene expression (*GS1, DREB2*, and *WCOR*) traits had positive loading on the main vector (Dim 1) for both LTR and STR+. Similarly, traits like anthesis time (flowering), water status (δ^13^C) and b^*^, as well as the expression of *TIP1.1* and *GS2* had negative loading in the vector lines (Supplementary Figure S4); while *RBCL* and *DREB1* showed the opposite loading. On the second main vector, the biomass traits were distributed across the X-axis and in the same quadrant, with no major difference between LTR and STR+ lines, except that *TIP1.1* had higher positive loading for LTR lines (Supplementary Figure S4), whereas it has lower weight in STR+. In the same way, SPAD had a strong positive loading for STR+ lines.

The physiological and water status traits were the most influential traits in both groups. The gene expressions of *RBCL, GS1, GS2*, and *TIP1.1* were highly influential in the LTR lines, while *DREB1, DREB2*, and *WCOR* were more influential in STR+ (Supplementary Figure S4). The gene expression of *GS1* relative to *GS2* and SPAD showed a close correlation with respect to Dim 2 in the LTR lines, while in the STR+ lines these traits were opposite, indicating their independence.

## Discussion

### The Transpiration Response of Wheat Lines Under Increasing VPD

The ability to segment the transpiration response or restrict somehow the water loss through the stomata, measured as the transpiration response to a variation in VPD in the vegetative stage, allowed us to classify 20 durum wheat lines into three significantly different groups (Table 3, Figure 1): linear transpiration response (LTR), less segmented transpiration (STR−) and very segmented transpiration (STR+). The LTR lines did not limit their transpiration as the VPD increased; furthermore their normal transpiration in the range 1–3 kPa was lower than the STR+ lines (Supplementary Figure S1). A linear pattern in the transpiration increase in response to rising VPD may be characteristic of elite wheat lines (in our case commercial lines), which may retain open stomata as the VPD increases (Schoppach and Sadok, 2012). Previous studies in wheat have also reported genetic variability in the transpiration response (Schoppach and Sadok, 2012, 2013). However, in contrast to these earlier studies, differences in the transpiration response to increased VPD were identified at relatively low VPD (around 1 kPa). Moreover, when the slope of the increase in transpiration was between 1 and 4 kPa, the groups of lines, as classified following the protocol published for wheat by Schoppach and Sadok (2012), did not exhibit any consistent differences in yields across the set of environmental conditions assayed. In addition, for the 20 commercial lines assayed we failed to find a break in the linear pattern of increase in transpiration as VPD rose above 1 kPa (Schoppach and Sadok, 2012; Schoppach et al., 2017). However, the same authors (Schoppach and Sadok, 2013; Schoppach et al., 2016) have also reported a linear pattern of increasing transpiration with VPD values similar to those (ca. 4 kPa) of our study. The reasons of measuring transpiration in plants at the vegetative, rather than during the reproductive stage, are diverse, including of methodological nature. Vegetative plants are much more homogeneous in structure than plants at later stages, where the ear and the supporting stem may represent a significant portion of the area and their transpirative pattern is quite different to that of leaf blades (Araus and Tapia, 1987; Araus et al., 1991; Tambussi et al., 2007). Moreover, conclusions from mature (e.g., at grain filling) plants growing in pots are not so straightforward since they may not represent the performance of the plants growing in the field and having a well-developed root system. There are also other practical reasons. Any phenotypic protocol to be useful for breeding have to be high throughput, which may be the case of seedlings growing under control conditions, for a limited number of days and under rather small pots. Moreover, the sense of phenotyping is to help predicting crop performance of genotypes, rather than to measuring the consequences of different agronomical performance, which may be the case if mature plants rather than seedling plants are measured. In fact, it is expected that genotypic differences in water management during vegetative stage will translate further to growth, biomass accumulation, and then grain yield. Accumulated biomass and grain yield are time-integrative traits. In this sense Nakhforoosh et al. (2016) reported that early growth shoot phenotyping is appropriate for the identification of water use strategies in a collection of durum wheat lines.

The STR- and STR+ groups of lines encountered the transpiration limitation (i.e., change in transpiration slope as VPD increase) at similar but significantly different breakpoint values close to 1 KPa. These VPD values are clearly lower than the 2 KPa or greater reported previously in wheat, which included lines selected for the Mediterranean conditions of Australia (Schoppach et al., 2017). However, there are reports indicating that stomatal closure may already start at mild VPD values below 2 kPa (Choudhary et al., 2013; Gholipoor et al., 2013; Choudhary and Sinclair, 2014). The mechanism that causes stomatal closure at high VPD is not well understood (Streck, 2003). The feedforward hypothesis states that stomatal conductance decreases directly as VPD increases, with abscisic acid (ABA) in the leaves probably triggering the response (Bunce, 1997, 1998). The feedback hypothesis states that stomatal conductance decreases as VPD increases because of an increase in transpiration (E) that lowers the leaf water potential. The results available for wheat are not consistent with stomatal closure at high VPD being a response to an increased whole leaf transpiration rate or lower leaf water potential. The lack of response of conductance to VPD in CO_2_-free air suggests that ABA may mediate the response (Bunce, 1998).

In wheat, previous reports have described variable adaptation strategies to maintain a stable photosynthetic surface while water is adjusted in response to transpiration demand (Schoppach and Sadok, 2012). Any negative relationship between transpiration rates and plant leaf area may suggest a trade-off between these traits. Other studies in wheat have reported either no correlation (Schoppach et al., 2017) or a negative correlation (Schoppach and Sadok, 2013; Schoppach et al., 2016). In our study we did not find a clear relationship between plant leaf area and transpiration (data not shown).

### Effect of the Transpiration-Response to VPD on Crop Performance Under a Range of Growing Field Conditions

The nine growing conditions assayed produced a wide range of grain yields under Mediterranean conditions, ranging from severely stressed to near optimal conditions (Acreche et al., 2008; Araus et al., 2013). Besides the grain yield, in order to characterize the different growing conditions and genotypic performance several physiological traits were measured during grain filling, since this is the final period, during crop cycle, in terms of yield setting. Such differences were associated with water status as shown by the lower g_s_ and CTD, together with the higher (less negative) δ^13^C of mature grains (Cabrera-Bosquet et al., 2011) in the less productive trials, while the better trials exhibited larger and greener canopies as indicated by the differences in spectroradiometrical and RGB canopy vegetation indices such as NDVI, Hue, lightness, a^*^, u^*^, v^*^, and GA (Casadessús et al., 2007; Elazab et al., 2015).

Evaluation of the set of modern durum wheat lines under a wide range of environmental growing conditions in the field confirmed that the LTR vs. STR groups of lines performed quite differently in terms of grain yield, depending on their growing conditions (Table 4 Low-Medium Yields and High Yields sections). Thus, in the trial with the lowest yield (Valladolid, 2014 rainfed, Table 4 Low-Medium Yields section), which was associated with water scarcity (the trial with the highest δ^13^C of grains), the STR lines had higher grain yields as well as higher vegetation indices (GA, NDVI, SPAD) than the LTR lines. Moreover, δ^13^C was higher in the STR than the LTR lines, which indicates a higher water use efficiency in the STR lines (Farquhar and Richards, 1984) that could favor the uptake, assimilation, and remobilization of N to the grains (Alva et al., 2006; Hirel et al., 2007).

In the seven trials with the highest yields and greatest water availability, the LTR lines showed greater yields and green biomass (NDVI, lightness, a^*^, and u^*^) (Table 4 High Yield section, Figure 2) than the STR lines. Moreover, in the most productive trial (Valladolid, 2015 irrigation), δ^13^C was more negative in the LTR compared to the STR lines and correlated negatively with grain yield across the set of 20 lines (R^2^ = −0.40, *p* < 0.05, data not shown), indicating that the more negative the δ^13^C the better the water status of the crop (Araus et al., 2003). In other studies in wheat under relatively good agronomical conditions negative correlations of δ^13^C with g_s_ and grain yield (Fischer et al., 1998; Lu et al., 1998) have been reported. In fact, it has been reported that apart from very drought-prone environments genetic advances in the yields of wheat and other species are related to a higher stomatal conductance (Roche, 2015 and references therein). Overall, the results show the existence of a line × environment interaction between the LTR and STR groups of lines. Thus, LTR lines exhibit higher grain yield, biomass and greenness under good to optimal agronomical conditions, whereas the STR lines perform better under the most water-limiting trials. The segmentation or limitations in the transpiration of STR lines due to their transpiration sensitivity fits with previous studies in other crop species concerning the water saving capacity of crops to enhance yield under severe water stress conditions (Kholová et al., 2010b, 2012; Belko et al., 2013; Vadez et al., 2014).

### Integration of Physiological Traits and Gene Expression in Response to Limitations to Water Loss Under Contrasting Yield Scenarios

**Figure 4:**
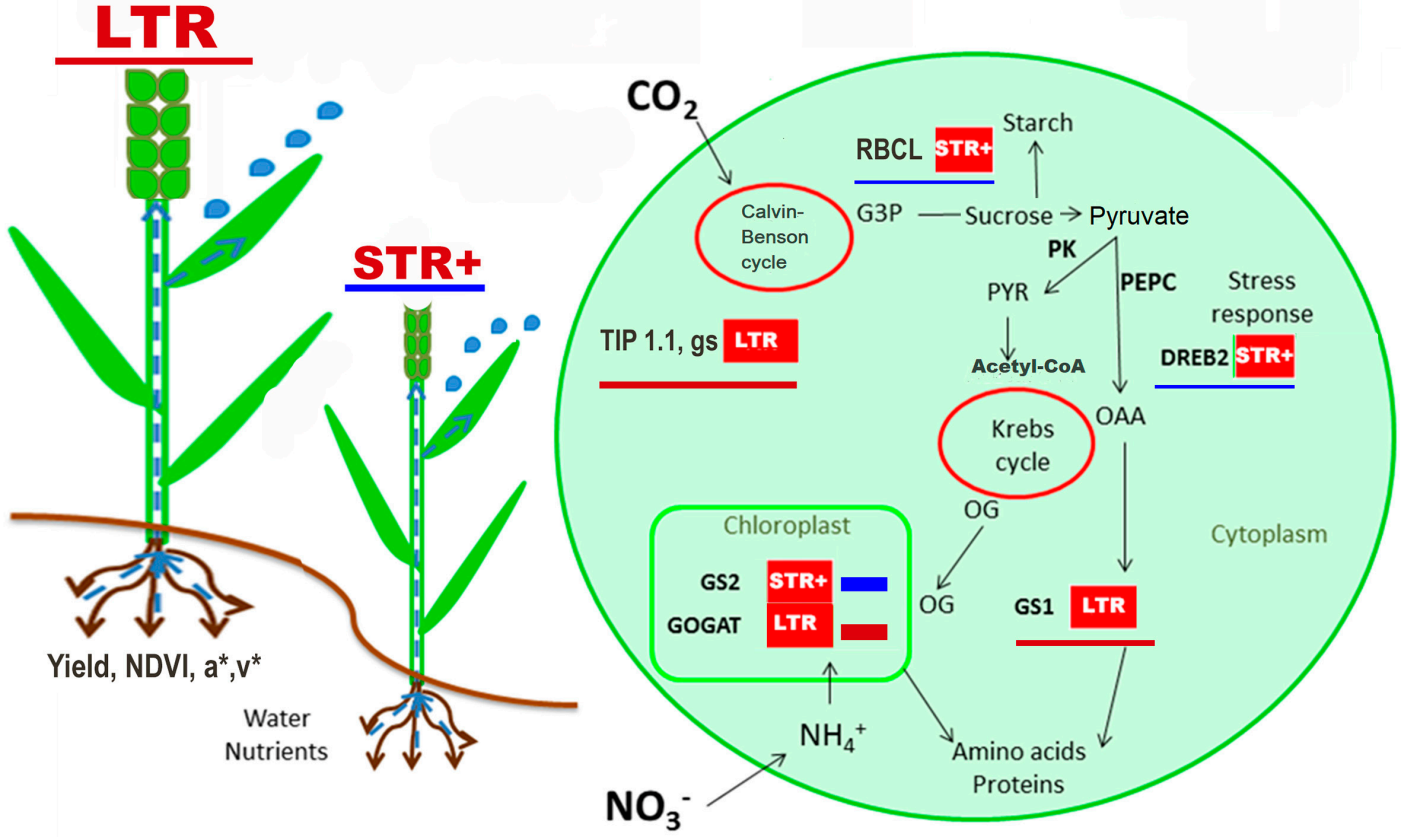
Overview of the changes in physiological traits and gene expression between very segmented transpiration (STR+) and linear transpiration (LTR) durum wheat lines. The scheme shows the significant mean expression of better or up-regulated values of LTR lines (red underline) and STR+ lines (blue underline) as well as significant traits evaluated across all field trials and the integrated pathway of N and C metabolism. Data used corresponds to the 20 lines growth in the different yield environments (i.e., trials) where gene expression was analyzed, with three biological replicates (i.e., three plots of each trial) for each line and yield environment. For trait and transcript abbreviations see Tables 4, 5.

Plant phenotype is based on the complex association between environment and physiological responses driven by gene regulation, which also determines growth and crop productivity. The integration of physiological traits with transcript profiles for genes involved in the response to stress and C and N metabolism may help to understand the adaptation strategies for a given environment (Kosová et al., 2014b). Our study showed patterns between the HY and LY scenarios and compared the STR+ and LTR groups (Figure 3), whose characteristics are probably driven by stress-responsive genes that influence expression of the basal metabolism and water transport genes described in Table 5. Significant differences in transcript expression suggest a major role for transcription factors (*DREB1* and *DREB2*) and dehydrins (*WCOR, DNH16*) influencing the expression of key genes in primary metabolism and water transport (*GOGAT, GS1, GS2, RBCL*, and *TIP1.1*). *DREB1* and *DREB2* seemed to be central to the integration of plant responses to the growing-conditions, and appeared to be co-regulated with water status traits (g_s_, CTD and δ^13^C) in both the LTR and STR+ groups (Figures 3A,B,D, 4).

*DREB1* was overexpressed in LTR compared to STR+ lines under high-yielding conditions (Table 5). *DREB1* may be a key regulator of metabolic signals in response to environmental conditions. It may drive the regulation of N remobilization and the provision of carbon skeletons for biomass development. Thus, this gene is positively correlated with genes involved in N metabolism (*GOGAT*), the provision of carbon skeletons (*PK* and *PEPC*), and the water status (δ^13^C and CTD) as well as canopy vegetation (lightness and v^*^ indices) (Figures 3A,B,D). An overexpression of *DREB1* may function as a protection signal against water scarcity, as reported in wheat and other cereals (Zhao et al., 2016), and is likely to positively influence the regulation of *GS1* as reported in relation to metabolic imbalances (Thomsen et al., 2014).

In the case of *DREB2* it was up-regulated in STR+ lines compared to LTR, especially under stress conditions (Table 5). Previous studies in durum wheat have reported an increase in *DREB2* in response to water and salinity stresses (Sheshadri et al., 2016; Yousfi et al., 2016). The close relationship of *DREB2* with water status traits (δ^13^C and g_s_), and *DNH16* dehydrin under low yield conditions (Figures 3D, 4 and Table 5) supports the concept that the up-regulation of *DREB2* in STR+ lines helps them to adapt to an inherently poorer water status. Such an assumption is supported by reports of overexpression of *DREB2*-type genes (*TaDREB2, TaDREB3*, and *TaDREB5*) in low yielding wheat lines (Morran et al., 2011; Shavrukov et al., 2016). Moreover, *DREB2* may interact with ABA signaling to drive root hydraulics and the transpiration response in wheat (Schoppach et al., 2016).

Dehydrin (*DNH16* and *WCOR*) expression was higher in high-yielding conditions, which contrasts with reports of enhanced dehydrin signals under drought conditions (Rampino et al., 2012). In high-yielding conditions the expression of DNH16 was positively correlated with the expression of genes encoding C (*PK*) and N (*GS2*) metabolism enzymes, which suggests that this dehydrin may play a protective role in ensuring greater N assimilation (*GS2*) and carbon skeleton transformation (*PK*) (Figures 3A,B). Moreover, our study suggests that the dehydrin response may also be driven by transcription factors; i.e., the negative relationship between *DNH16* and *DREB2* agrees with the work of Kosová et al. (2014a). Similarly, WCOR overexpression, which was positively correlated with the expression of *ATPase* (Figure 3A), matches other reports of overexpression of *WCOR* genes in high-yielding wheat and barley lines (Tsvetanov et al., 2000), as well as its regulatory function in stomatal opening (Danyluk et al., 1996).

Concerning the N metabolism genes (Table 5 and Figure 4), GS2 transcripts were significantly higher under low-yielding than high-yielding conditions, reflecting the greater need for N assimilation under unfavorable conditions. The *GS1* and *GOGAT* genes were down-regulated in STR+ compared to LTR lines (Figure 4); this pattern suggests a lower N remobilization in the STR+ lines due to their lower yield. Thus, the *GS1* overexpression in flag leaves of LTR lines compared to STR+ lines under optimal conditions may suggest a better capacity of the former to remobilize N from the leaves to grains. Recent results in wheat have concluded that higher *GS1* transcripts support N remobilization to the grains (Zhang et al., 2017) as well as a higher nitrogen use efficiency (Thomsen et al., 2014; Tian et al., 2015). In agreement with this, we found a higher grain nitrogen yield in the LTR lines than the STR+ lines (for example 180 vs. 174 kg ha^−1^ in Valladolid support irrigation 2014 and 154 vs. 144 kg ha^−1^ in Aranjuez support irrigation 2014, respectively). In the case of GS2, grain nitrogen yield was upregulated in STR+ lines, particularly under high-yielding conditions, which may indicate a need for N assimilation due to a clear inhibition of N remobilization and/or a low content of end products.

RBCL was significantly over-expressed in STR+ lines compared with LTR lines under high-yielding conditions. The good agronomical conditions favored the LTR lines in terms of yield, which may imply less of a need to increase the capacity for photosynthetic CO_2_ fixation and may benefit plant growth by diversifying the high amount of N invested in Rubisco. In that regard, Rubisco upregulation in STR+ lines was accompanied by overexpression of *GS2*. This was probably a response to a higher demand for N supply to synthesize more Rubisco enzyme, which agrees with previous reports about co-ordinated regulation of CO_2_ fixation and N assimilation during grain filling in wheat (Nagy et al., 2013; Komatsu et al., 2014) and especially in durum wheat (Vicente et al., 2015).

On the other hand, under low yield environments the downregulation of the *RBCL* gene in the STR+ lines relative to the LTR lines may be just the consequence of the better growing capacity of the former. A small decrease in Rubisco expression can lead to an improvement in biomass and grain yield due to lower N allocation to Rubisco synthesis, and greater investment in other limiting processes, as described for rice (Kanno et al., 2017).

The aquaporin *TIP1.1* was significantly upregulated in LY compared to HY scenarios and was greatly influential in the LTR lines (Table 5 and Figure 4), which agrees with the role of TIP1.1 in favoring water channel activity under low water availability conditions (Tardieu et al., 2014), and therefore cell rehydration (Willigen et al., 2004). This aquaporin expression pattern, together with the upregulation of *GS2*, which also increases under LY compared with HY conditions (Table 5), may also favor N assimilation. The *TIP1.1* gene in durum wheat is homologous to the HvTIP1;1 gene in barley, which has been demonstrated experimentally as transporting water as well as urea and hydrogen peroxide (Hove et al., 2015). The *TIP1.1* gene was underexpressed and had lower influence on the STR+ compared to the LTR lines (Table 5 and Figure 4), supporting different mechanisms of water transport between the two groups. The higher *TIP1.1* expression in LTR lines may be associated with a higher stomatal conductance and transpiration, alongside a lower δ^13^C, and this is eventually associated with a higher water use and grain yield. These results are indicative of a favorable role for aquaporins in plant water transport through control of hydraulic conductance, therefore favoring water transport and photosynthesis (Sade et al., 2009; Moshelion et al., 2015), which influences plant metabolism (Vera-Estrella et al., 2004; Forrest and Bhave, 2007) and increases diffusion of CO_2_ probably due to a better control of the stomata (Kawase et al., 2013).

An overview of general physiological and transcriptional switches between STR+ and LTR lines (irrespective of the yielding environment) (Figure 4) showed that higher transpiration capacity in LTR lines seems to be associated with or influenced by higher aquaporin expression, suggesting better water transport. Further, the LTR group showed better biomass (NDVI) and greenness (a^*^ and v^*^), which may also be associated with a more efficient distribution of N within the plant as it is suggested by the *GS1* overexpression. This is in line with the importance of GS enzymes for yield production (Martin et al., 2006; Yousfi et al., 2016). The LTR lines exhibited not only higher productivity but also higher biomass and nitrogen grain yield, except for the most stressed environments; in these lines the aquaporins play an instrumental role. Also, the over expression and greater influence of DREB2 in the STR+ lines may play a positive role at the leaf level under severe water stress conditions, where it is associated with increasing chlorophyll content. Moreover, over expression of DREB2 has been reported to impact positively nitrogen assimilation and photosynthetic carbon fixation and probably limits the water loss by the plant (Salekdeh et al., 2009). This fits with a recent study in wheat where a major QTL was reported to control the transpiration response to VPD (Schoppach et al., 2016). This QTL harbored several genes involved in ABA signaling and the interaction of ABA with *DREB2A* and root hydraulics.

## Conclusions

This study provides evidence of how the ability of wheat lines to limit or not limit transpiration during vegetative stage may affect agronomical performance at further stages of the crop cycle and under a wide range of environmental conditions in the Mediterranean. Thus, LTR lines performed better in terms of yield than the STR lines, with the exception of the very dry environmental conditions. Moreover, the study highlights the complexity of physiological and molecular mechanisms associated with these different transpiration responses to high VPD. At the gene expression level both groups of lines are regulated by DREB transcription factors and dehydrins. However, the results suggest that the higher grain yields of LTR lines is in line with their superior water status, and in turn this is associated with more active aquaporins and specific adaptations to carbon and nitrogen metabolism driven by regulation of genes that encode key enzymes. The negative but marginal correlation between plant leaf area and leaf transpiration suggests that the trade-offs between these traits are minor and supports further studies to explore the feasibility of using transpiration for selecting wheat lines better adapted to Mediterranean conditions.

## Author Contributions

SM and JA designed the experiments. SM conducted the experimental work. MN-T and NA grew the field crops during both seasons. SM, RV, MN-T, NA, FC, and OV-D recollected the field data. SM, RV, and JA contributed to the data analysis and interpreted the results. SM wrote the paper under the supervision of RV and JA. All authors revised the manuscript and read and approved the final version.

### Conflict of Interest Statement

The authors declare that the research was conducted in the absence of any commercial or financial relationships that could be construed as a potential conflict of interest.
