# Supplementary material for: The Plant-Transpiration Response to Vapor Pressure Deficit (VPD) in Durum Wheat Is Associated With Differential Yield Performance and Specific Expression of Genes Involved in Primary Metabolism and Water Transport
Source: Front Plant Sci. 2019 Jan 15;9:1994. doi: 10.3389/fpls.2018.01994 (PMC6341309; doi:10.3389/fpls.2018.01994)
Supplement: Supplementary file 1 [file Table_1.pdf]

frontiers

Supplementary Material

The Plant-Transpiration Response to Vapour Pressure Deficit (VPD) in Durum Wheat is Associated with Differential Yield Performance and Specific Expression of Genes involved in Primary Metabolism and Water transport

Susan Medina[1,2], Rubén Vicente[1], Maria Teresa Nieto-Taladriz[3], Nieves Aparicio[4], Fadia Chairi[1], Omar Vergara-Diaz[1] and José Luis Araus[1*]

[1]Integrative Crop Ecophysiology Group, Plant Physiology Section, Faculty of Biology, University of Barcelona (UB), Barcelona, Spain.

[2]Universidad Científica del Sur, Lima, Perú.

[3]National Institute for Agricultural and Food Research and Technology (INIA), Madrid, Spain

[4]Agricultural Technology Institute of Castilla and León (ITACYL), Valladolid, Spain

* Correspondence: Dr. Jose Luis Araus: jaraus@ub.edu

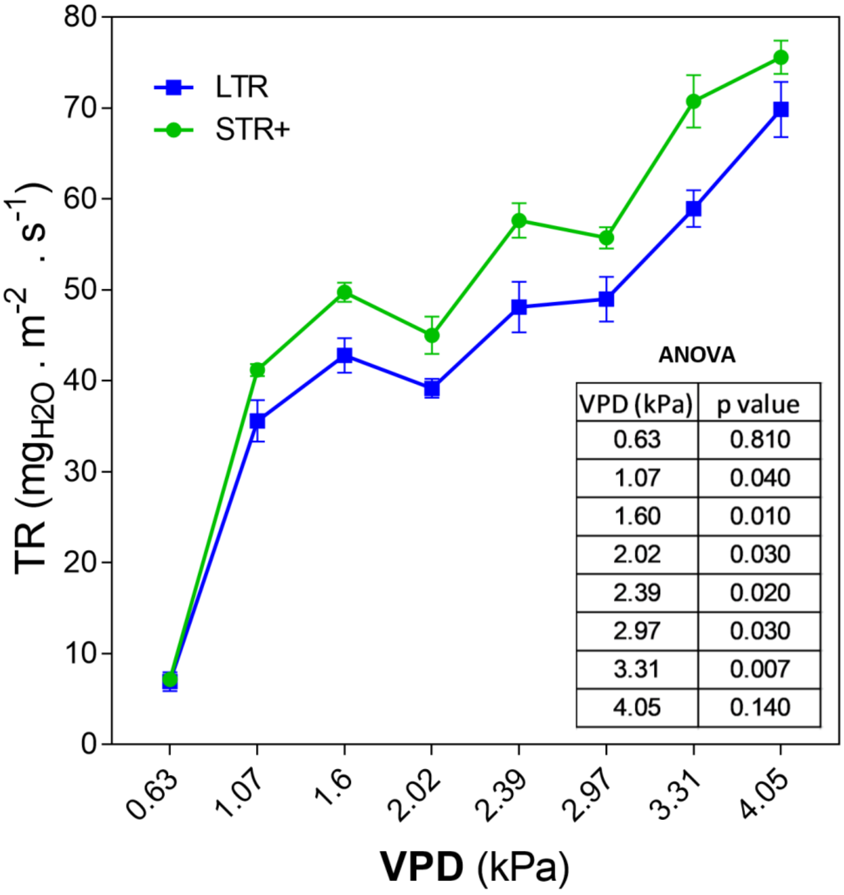

Supplementary Fig. S1. Transpiration rate (TR) of the linear transpiration (LTR, blue) and very segmented transpiration (STR+, green) subsets of the durum wheat lines exposed to increasing VPD from 0.63 kPa to 4.05 kPa. Each point expresses the mean transpiration rate of the groups at each VPD value in the range 1.07 kPa to 4.1 kPa, and the chart shows the ANOVA comparison between both groups at each VPD point. Plants were tested at the vegetative stage. While each point represents the mean value of any of the two different transpiration groups of lines, values for each individual line were calculated from five biological (i.e. pot) replicates.

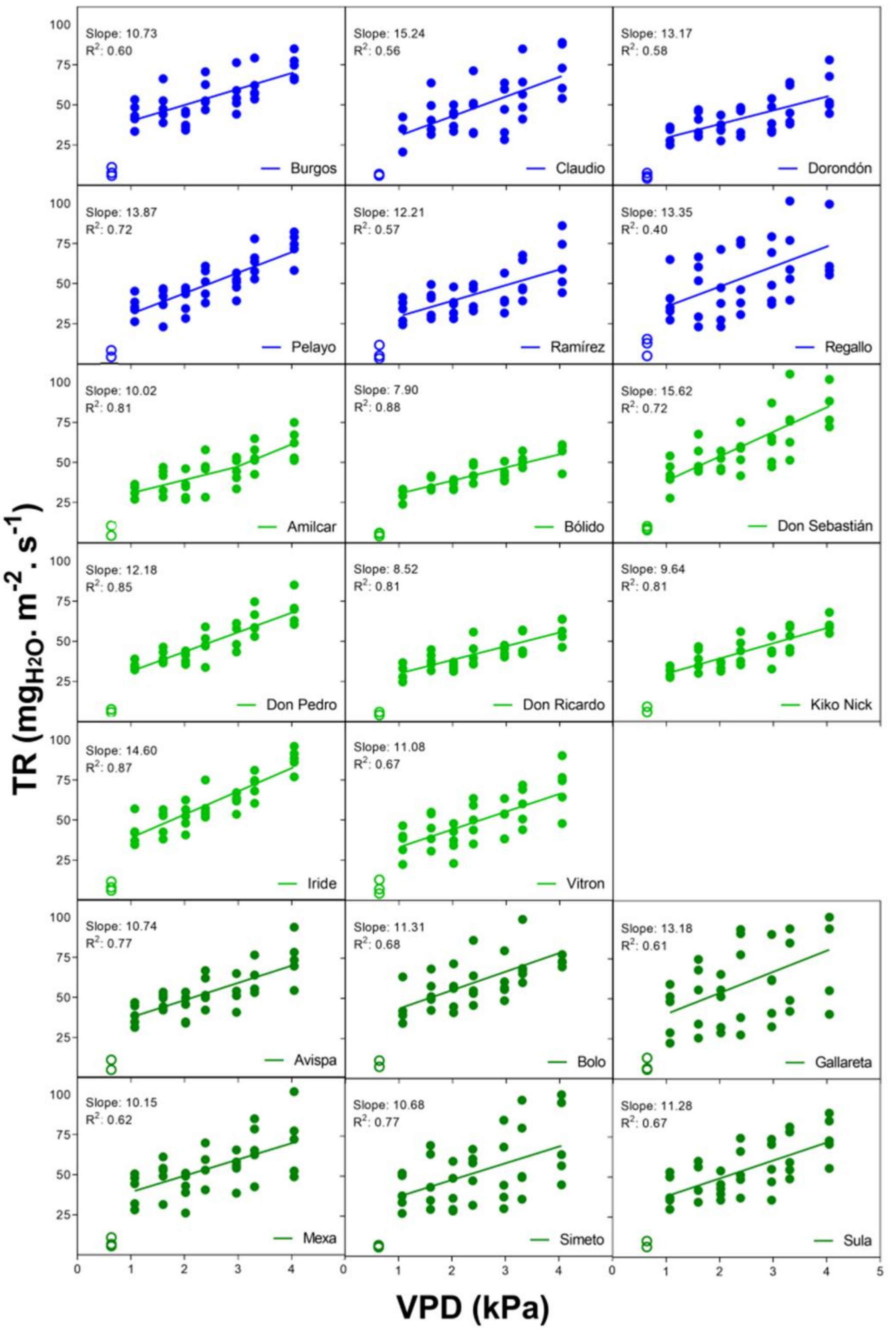

Supplementary Fig. S2. Transpiration rate (TR) of each of 20 durum wheat lines exposed to increasing VPD from 0.6 kPa to 4.1 kPa. Each curve expresses the linear regression between TR and VPD values in the range 1.07 kPa to 4.1 kPa (full circles), and the TR values at 0.6 kPa (empty circles) for each durum wheat line. Plants were tested at the vegetative stage and each symbol represents the average of five biological (i.e. pot) replicates. All panels show the mean slope of the linear regression and the R^2 value.

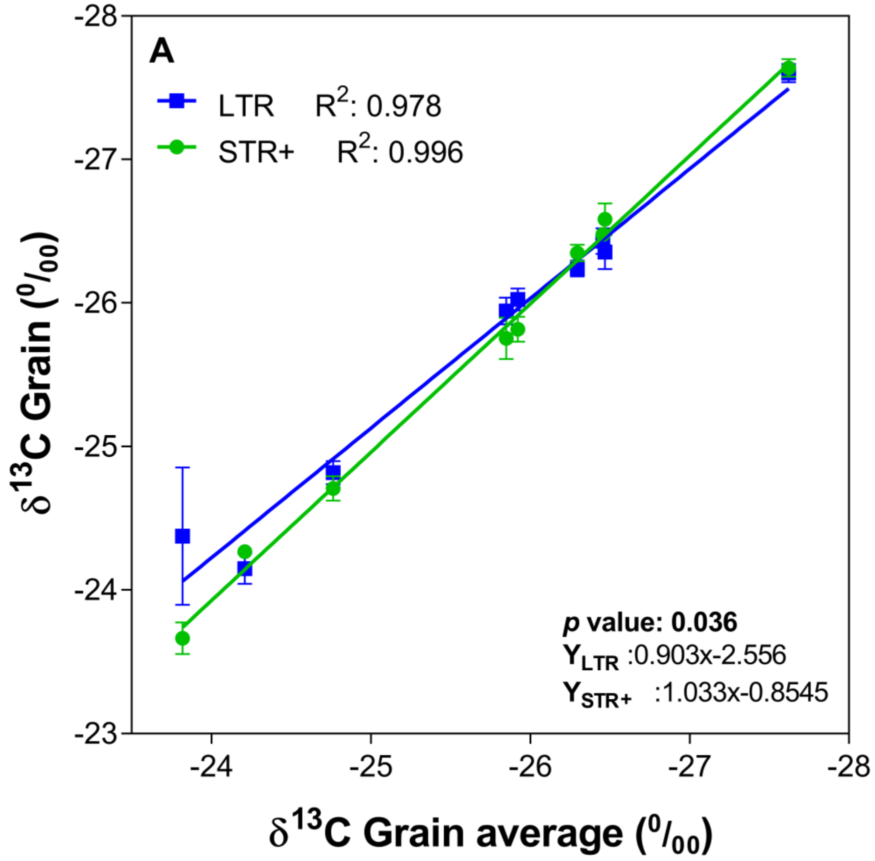

Supplementary Fig. S3. Relationship between grain carbon isotope composition (δ^{13}C) and grain yield between the very segmented transpiration (STR+) and linear transpiration (LTR) durum wheat lines. The graph shows the linear regressions of the average grain δ^{13}C of LTR and STR+ groups versus the average δ^{13}C value for the whole set of 20 lines evaluated in each one of the nine field scenarios. Each symbol represents the mean value of any of the two different groups of lines, with each line being the average of three different biological (i.e. plot) replicates. The fitted curves on the figure were significant (p<0.001). The level of significance (p) between fitting lines as well as the determination coefficient (R^2) and the equation of each line are also indicated.

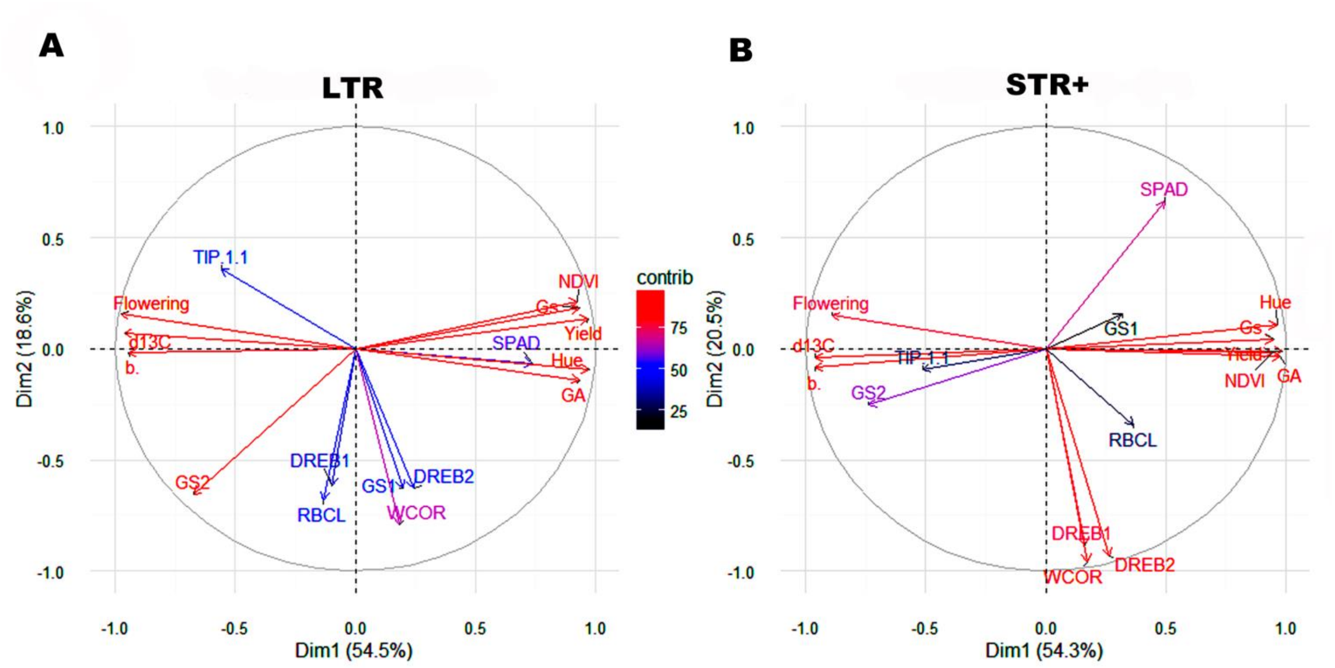

Supplementary Fig. S4. M**ultivariate analysis of the changes in physiological traits and gene expression between very segmented transpiration (STR+) and linear transpiration (LTR) durum wheat lines**. A and B show the principal component analysis (PCA) explaining the influence of physiological traits and gene expression for both groups of lines (LTR and STR+) under all growing conditions. The contribution of each trait corresponds to the colour scale (Contrib) and length of the vectors. For trait and transcript abbreviations see Tables 3 and 4; Flowering represents the anthesis time, and b. is the b* vegetation index. Except for the carbon isotope composition ($\delta^{13}C$) of mature kernels, the rest of the physiological and gene expression traits were evaluated during the reproductive stage in the field. For each of the two groups of lines, values of individual lines at each of the growing conditions (trial) were calculated as the average of three different biological replicates.

TABLES

Supplementary Table S1. Different categories of measurements performed in controlled and field conditions. For each line, traits under controlled conditions were measured in five different biological replications, while field traits (either agronomic, physiological or molecular) were measured in three biological replicates per growing condition (trial).

Controlled conditions (Exp. 1)	Field conditions (Exp. 2)	Molecular laboratory (Field samples)
Transpiration rate<br>Dry biomass<br>Leaf area	Spectroradiometrical vegetation indices: NDVI<br>Leaf chlorophyll content: SPAD<br>RGB (red-green-blue) vegetation indices: hue, intensity, saturation, lightness, a*, b*, u*, v*, and GA<br>Water status: stomatal conductance (g_s), canopy temperature (CTD), carbon isotope composition ($\delta^{13}C$)<br>Nitrogen status: total nitrogen content and nitrogen isotope composition ($\delta^{15}N$)<br>Phenology (anthesis time)<br>Grain yield	RNA extraction<br>RNA quantification<br>RNA integrity<br>cDNA synthesis<br>Real time PCR<br><br>Genes: *DREB1*, *DREB2*, *DHN16*, *WCOR*, *SOD*, *ATPase*, *GS1*, *GS2*, *GOGAT*, *PEPC*, *PK*, *RBCL* and *TIP1.1*.

Supplementary Table S2. Set of modern (semi dwarf) durum wheat cultivars tested in this study. Year of release, country of registration, pedigree or origin are presented with the period during which each variety was considered among the main cultivated varieties in Spain and the maximum and minimum percentage of area occupied during this period, Plant height (PH) and days to heading (DH).

Variety	Year of release	Country	Pedigree/cross name or origin	Period[a]	Max[a]	Min[a]	PH (cm)	DH
Mexa	1980	Spain	GERARDO-VZ-469/3/JORI(SIB)//ND-61-130/LEEDS	1993-2004	14.9	1.5	93.49	144
Vitron	1983	Spain	TURCHIA-77/3/JORI-69(SIB)/(SIB)ANHINGA//(SIB)FLAMINGO	1993-2011	34.0	3.1	88.68	147
Regallo	1988	Italy	Diputación General de Aragón CIMMYT	1995-2012	8.2	2.6	82.16	148
Simeto	1990	Spain	RUFF/FLAMINGO//MEXICALI-75/3/SHEARWATER	1993-2010	15.3	1.2	83.08	148
Gallareta	1994	Spain	CIMMYT	1993-2012	10.5	3.3	89.31	148
Bolo	1994	Spain	CARCOMUN/AUK	1998-2005	4.2	0.8	92.8	154
Don Pedro	1994	Spain	SHEARWATER(SIB)/(SIB)REDNECK//(SIB)YAVAROS	2001-2010	4.4	0.6	88.73	148
Sula	1994	Spain	CIMMYT	1993-2010	4.3	0.5	84.56	148
Bólido	1996	Italy	STN'S'//HUI'S'/SOMO'S'	2007-2010	2.3	1.0	83.24	149
Dorondon	1998	Spain	Genética y Gestión,S.C.	2004-2010	4.5	0.6	90.41	147
Iride	1998	Italy	Altar 84 × Ares sib	2001-2012	7.3	0.4	84.77	147
Burgos	1999	Spain	SUDDEUTSCHE SAATZ	2003-2012	5.3	1.0	89.7	150
Claudio	1999	Spain	(Sel. Cimmyt × Durango) × (IS193B × Grazia)	2008-2012	3.6	0.5	91.64	149
Amilcar	2001	Italy	ZEGZAG-1/LUNDE-5//GREENSHANK-32	2005-2012	10.6	2.0	83.39	146
Pelayo	2002	Spain	CAPEITI-8/VALNOVA	2005-2012	16.9	0.4	84.44	147
Avispa	2003	Spain	Limagrain-CIMMYT	2009-2010	1.2	0.5	84.48	146
Don Sebastián	2004	Spain	Agrovegetal-CIMMYT	2005-2010	5.6	3.6	97.71	149
D Ricardo	2008	Spain	Agrovegetal-CIMMYT	2008-2012	5.0	0.5	91.03	149
Kiko Nick[b]	2009	Spain	SEL.CIMMYT-35/DURANGO//ISEA-1938/GRAZIA				88.09	147
Ramirez[b]	2009	Spain	ALTAR-84/IONIO				89.67	152

(a) data from Asociación Española de Técnicos Cerealistas (AETC) 1992/93 to 2011/12

(b) high yielding cultivars in the Spanish national trials (GENVCE)

Supplementary Table S3. Primers for the housekeeping and target genes used for qRT-PCR analysis. Genes assayed and their names and sequence accession numbers are shown. The right column indicates the forward (F) and reverse (R) sequences of primers mentioned in the Materials and Methods.

Gene	Name	Sequence 5´-3´
	Housekeeping genes	
18S	18S ribosomal subunit (M82356)	F: GGCCGCTCCTAGCCCTAATTG<br>R: TGAGCACTCTAATTTCTTCAAAGTACG
UBI	Ubiquitin (Ta50503)	F: GCACCTTGGCGGACTACAACATTC<br>R: GACACCGAAGACGAGACTTGTGAACC
	Target genes	
TIP1.1	Aquaporin *TIP1.1* (EU177566)	F: TGAGTTCCTTCTTCCTTCCTTCTTC<br>R: TTTTTGCCCTGTCCTGTCGTAG
DREB1	Transcription factor *DREB1* (AF303376)	F: CACTCTCTTGGATGGTAGTGTCG<br>R: GTGTATTCTCAGGTCCTCCTTTCC
DREB2	Transcription factor *DREB2B* (AB193608)	F: CTCTGAAACGATCAGGCGATGG<br>R: GTGTATTCTCAGGTCCTCCTTTCC
SOD	Superoxide dismutase (KP696754)	F: GGGTGTGGCTAGCTTTGGAT<br>R: TGCAGGTTTGACCCTTTGGT
WCOR	Actin-binding protein *WCOR719* (U58278)	F: TTCTTCATCCACTGGTCGCC<br>R: GGAGCTGGCATACAGCATCT
GOGAT	Ferredoxin-dependent glutamate synthase (TC394038)	F: CGGCAATGGAGGCTGAGCAACA<br>R: TGAGCCTGCTCGATGGTCACTGT
DHN16	Dehydrin *Td16* gen (X78429)	F:aCGAGGCCAAGCACAAGG<br>R: TCTGCTTGGTCGTCTCCG
GS1	Cytosolic glutamine synthetase (DQ124209)	F:aAGGACGGCGGGTTCAA<br>R: GCGATGTGCTCCTTGTGCTT
GS2	Chloroplastic glutamine synthetase (DQ124212)	F: GATGGAGGTTTCGACGTGAT<br>R: CAAGTCAGGCGAAGTGAAA
PEPC	Phosphoenolpyruvate carboxylase (Y15897)	F: CAGACTGGCGAGCTCTTCTT<br>R: GACGAAGCGTGGTTCTTGGA
PK	Pyruvate kinase (AK332778)	F: CCATGCTTGCCGATCCACGTCA<br>R:aCGACAACGCGGTCATGCGA
ATPase	Chloroplastic ATP synthase β-subunit (M16843)	F: CCCTGCCCCTGCCACAACATTT<br>R: GTTGCCAACGATCCGAGGCTGT
RBCL	Rubisco large subunit (KM668209.1)	F: CGTGCTCTACGTTTGGAGGA<br>R: TTGGATACCATGAGGCGGG

Supplementary Table S4. Linear adjustment of the transpiration response (TR) to variations in vapour pressure deficit (VPD) in 20 durum wheat lines. The lines were fitted to linear regressions (p<0.001) for the transpiration response during changes in VPD between 1.07 kPa and 4.1 kPa. The values represent the mean of five biological replicates. The parameters evaluated are the slope of TR vs. VPD with its R^2 of the fitted curve, and the TR at 1.07kPa, 2.02 kPa and 4.1 kPa. At the bottom is shown the average comparison between non-restrictive (NR) and restrictive (R- and R+) lines according to the LSD test (p<0.05). The TR is expressed as $mg_{H2O}\ m^{-2}\ s^{-1}$ and the slope in $mg_{H2O}\ m^{-2}\ s^{-1}\ kPa^{-1}$.

	Class	*Line*	*Slope*	*R^2*	*TR (1.07 kPa)*	*TR (2.02 kPa)*	*TR (4.1 kPa)*
**Linear transpiration (LTR)**	LTR	Burgos	10.73	0.604	43.90	39.66	73.93
	LTR	Claudio	15.24	0.565	30.81	41.82	72.84
	LTR	Dorondón	13.17	0.576	29.68	35.49	58.35
	LTR	Pelayo	13.87	0.718	35.87	39.94	73.17
	LTR	Ramírez	12.21	0.575	33.02	36.73	63.08
	LTR	Regallo	13.35	0.403	40.27	41.42	77.70
**Segmented transpiration (STR)**	STR -	Amilcar	10.02	0.814	31.21	34.54	61.71
	STR -	Bólido	7.90	0.881	30.43	36.39	55.75
	STR -	Don Ricardo	8.52	0.811	31.05	33.89	56.64
	STR -	Don Pedro	12.18	0.855	34.45	38.98	69.84
	STR -	Don Sebastián	15.62	0.718	42.17	49.08	90.30
	STR -	Iride	14.60	0.872	42.74	52.12	87.82
	STR -	Kiko Nick	9.64	0.814	31.57	34.99	61.97
	STR -	Vitron	11.08	0.669	35.83	37.08	70.74
	STR +	Avispa	10.74	0.771	39.30	43.54	73.88
	STR +	Bolo	11.31	0.685	43.87	54.31	80.96
	STR +	Gallareta	13.18	0.614	42.03	46.69	81.07
	STR +	Mexa	10.15	0.62	40.64	41.64	70.50
	STR +	Simeto	10.68	0.773	40.09	40.39	72.29
	STR +	Sula	11.28	0.676	41.22	43.48	74.76
	**Linear transpiration**	**LTR**	**13.09 a**		**35.59 b**	**39.18 b**	**69.85 a**
	**Segmented transpiration**	**STR-**	**11.20 ab**		**34.93 b**	**39.64 b**	**69.34 a**
		**LTR+**	**11.22 b**		**41.19 a**	**45.01 a**	**75.58 a**
